# The human ABCG2 transporter engages three gates to control multidrug extrusion

**DOI:** 10.1016/j.isci.2025.112125

**Published:** 2025-02-28

**Authors:** Narakorn Khunweeraphong, Karl Kuchler

**Affiliations:** 1 Medical University of Vienna, Max Perutz Labs Vienna, Center for Medical Biochemistry Campus Vienna Biocenter, Dr. Bohr-Gasse 9/2, 1030 Vienna, Austria

**Keywords:** Natural sciences, Biological sciences, Biochemistry, Microbiology, Molecular microbiology

## Abstract

The human ABCG2 transporter plays roles in physiological detoxification across barriers and in anticancer multidrug resistance. The translocation pathway for drug extrusion and its gating mechanism remains elusive. Here, we demonstrate that the ABCG2 multidrug transporter holds two cavities that are delineated by three regulatory gates, indicating a substrate translocation channel. Drugs are trapped in the central cavity after entering through the pivotal intracellular entry gate. This flexible cavity is surrounded by a cluster of three highly conserved phenylalanines. Their aromatic side chains enact a “clamp-push-seal” motion to ensure unidirectional substrate movement. The unique residues T435 and N436 act as critical selectors for ligands, determining the broad substrate specificity. The upper cavity is covered by the lid architecture, constituting the final gate before multidrug extrusion. This work unravels deep mechanistic details on how the translocation channel utilizes pivotal gating steps, including the sequence of events that drive ABCG2-mediated multidrug efflux.

## Highlights

• The human ABCG2 multidrug transporter engages three gates to control substrate efflux
• A negatively charged residue at the first entry gate is indispensable for drug access
• Aromatic residues in the central cavity act as “clamp-push-seal” arms to drive drug extrusion
• Residues T435 and N436 are essential for both substrate and inhibitor selection

## Graphical abstract

**Figure:**
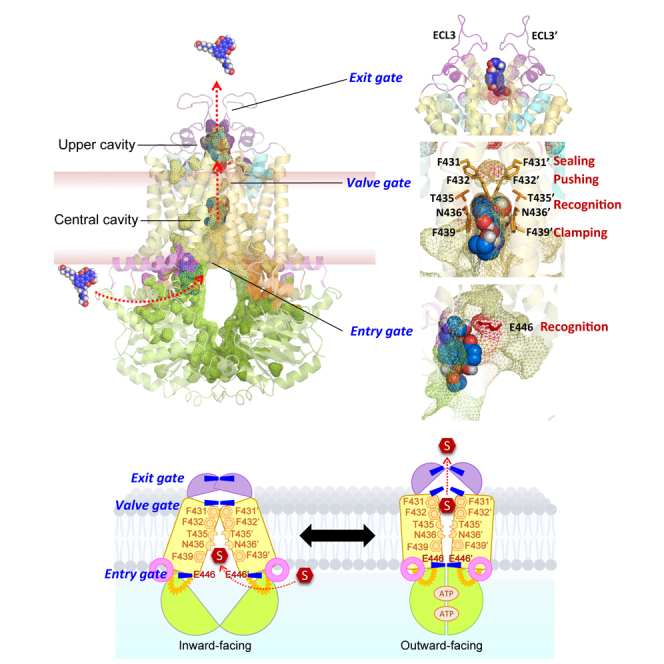

## Teaser

Physiology; Natural sciences; Biological sciences; Biochemistry; Genetics; Molecular Biology; Microscopy; Pharmacology; MD Simulations; Molecular Docking; Homology Modeling; Microbiology; Molecular microbiology

## Introduction

ATP-binding cassette (ABC) transporters represent a ubiquitous family of membrane proteins present in all living organisms. They transport a bewildering range of diverse molecules across membranes in an ATP-dependent manner.^1^^,^^2^ Some 48 human genes were originally classified into seven subfamilies (A to G).^3^ A more recent classification groups ABC proteins based on structural hallmarks.^4^^,^^5^ Three ABC transporters, ABCB1 (P-glycoprotein, P-gp), ABCC1 (multidrug resistance protein 1, MRP1), and ABCG2 (breast cancer resistance protein, BCRP), are key players in multidrug resistance (MDR) phenomena in cancer and microbial infectious diseases.^6^^,^^7^^,^^8^^,^^9^^,^^10^^,^^11^^,^^12^^,^^13^^,^^14^^,^^15^

ABCG2 is a half transporter that homodimerizes to form a functional export pump, adopting a unique fold as a type II multidrug exporter^16^ that recognizes over 130 substrates with a similar number of inhibitors as documented in Drugbank (https://go.drugbank.com). Its architecture includes an intracellular N-terminal nucleotide binding domain (NBD), followed by the elbow helix that parallels the inner membrane leaflet. At the transmission interface, ABCG2 has a highly conserved triple helical bundle (THB) formed by the NBD-elbow-ICL1 (intracellular loop 1) cluster.^17^ The transmembrane domain (TMD) consists of six transmembrane-spanning helices (TMHs), with the re-entry helix at the outer membrane leaflet. The extracellular loop 3 (ECL3) forms a lid-like roof structure at the cell surface.^18^ Interestingly, the closest homologues, ABCG5/ABCG8, ABCG1, and ABCG4, have a superimposable structure but exhibit a narrow substrate specificities limited to sitosterol (for ABCG5/ABCG8)^19^^,^^20^ and cholesterol.^21^^,^^22^ Thus, the precise molecular mechanisms by which each G family transporter effluxes diverse substrates remain as a conundrum. Notably, a point mutation (R482G) in the third TMH of ABCG2 expands substrate specificity, thereby increasing the overlap with ABCB1 and ABCC1.^23^^,^^24^^,^^25^ Further, the G185V mutation near TMH3 of human ABCB1 also enhances colchicine resistance, demonstrating a high sensitivity of drug binding to perturbations in transmembrane regions.^26^^,^^27^

To date, 25 ABCG2 cryoelectron microscopy (cryo-EM) structures at various resolutions and in diverse conformational states are available, 16 of which show a single substrate binding pocket in or at the central cavity.^16^^,^^28^^,^^29^^,^^30^^,^^31^^,^^32^^,^^33^^,^^34^^,^^35^ While the concept of gating mechanisms has been proposed for other membrane proteins, including P2-type ATPase sodium-potassium pumps,^36^ or the O antigen ABC transporters,^37^ none of the available ABCG2 structures discuss the existence of a gating mechanism. A gating step at the regulatory valve of ABCG2 was previously reported.^18^

Molecular docking has been used to yield new biological insights by predicting interactions between ligands and transporters,^38^ thus supporting the identification of molecular mechanisms. Here, we take advantage of ABCG2 structural models to generate 3D representations of the full-length ABCG2. We employ structural modeling in combination with molecular docking and extensive site-directed mutagenesis. Structural predictions and dynamic changes during the efflux cycle were validated by functional testing of mutant ABCG2 transporters using distinct drug substrates and inhibitors.

Our data reveal a molecular mechanism underlying drug extrusion of ABCG2 that relies on a highly dynamic “breathing architecture” within the transport pore, stretching from the inner membrane leaflet to the extracellular roof. This pathway features two major flexible cavities, the central and upper cavities, separated by three distinct regulatory gates, the entry gate, valve gate, and exit gate. The channel suggests a unidirectional route for drug extrusion, initiated by drug access at the entry gate(s), followed by recognition and trapping within binding regions of ABCG2. Substrates are classified by a unique selector before passing through the valve gate to reach the upper cavity, the final station before release into the extracellular space. We identify highly conserved aromatic residues as well as two ABCG2-unique residues that contribute to binding sites and regulate the translocation channel. Biochemical assays of ABCG2 mutant variants confirm the essential residues that determine selectivity and function, which are also controlled by three gating steps. These findings provide mechanistic insights into ABCG2’s broad substrate specificity and enhance our molecular understanding of the entire translocation pathway. This work offers valuable mechanistic insights into the structural dynamics of ABCG2, paving the way for novel drug development strategies to combat anti-cancer resistance.

## Results

### The ABCG2 translocation pore holds three gates that control drug efflux

**Figure 1:**
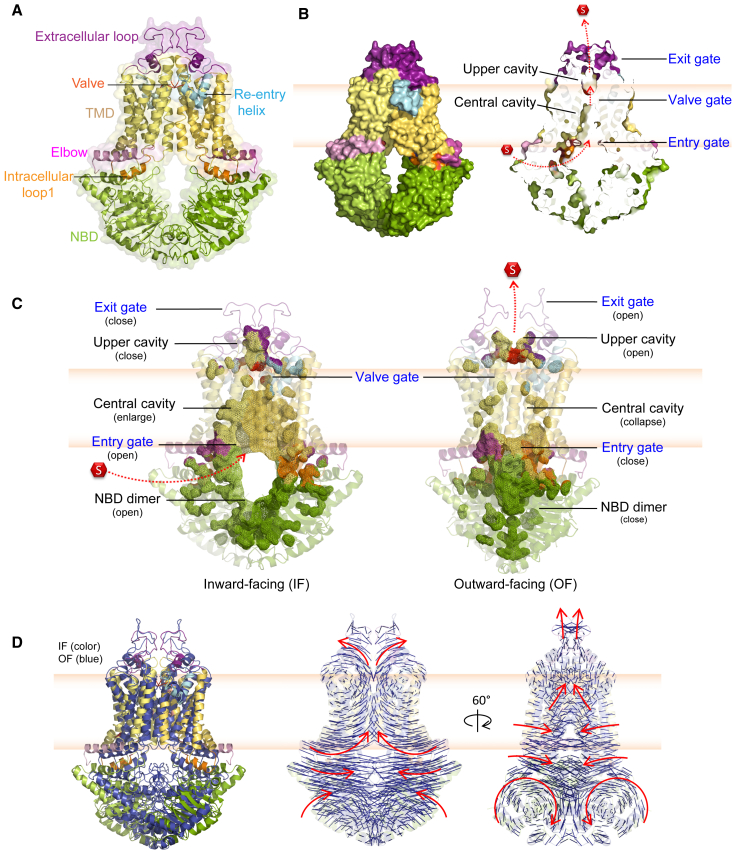
Architecture of the inner translocation path engages a triple-gated structure (A) The homodimeric human ABCG2 transporter in an inward-facing (IF) conformation (PBD ID: 6VXI) is shown in ribbon architecture with transparent surface rendering. Nucleotide binding domain, NBD (green), elbow helix (pink), first intracellular loop, ICL1 (orange), transmembrane helices (yellow), re-entry helices (cyan), leucine valve (red), ECLs (purple), and substrate (red hexagon). Consistent coloring shows the first half monomer in lighter tones and the second half is in a darker tone. (B) Side-view of ABCG2 structure in surface mode (left panel) and in the longitudinal cross-section along the molecule(right panel) reveals a translocation channel of ABCG2 with two distinct cavities, the central and the upper cavities, separated by three gates referred to as entry, valve and exit gates. (C) Drug extrusion drives massive structural changes of internal cavities that build-up the transporter core during the switch of IF to outward-facing (OF) states. The internal cavities and pockets are depicted in wireframe representation with corresponding colors as in (A) using transparent ribbon cartoons. (D) The dynamics of ABCG2 conformational changes during the drug transport cycle (residue to residue) in both orientations are indicated as the thin blue lines. Left: structural alignment between ABCG2 in an IF (colored code ribbon) with an OF states (blue ribbon). Middle and right: the red arrows indicate the directions of structural movements. Two NBDs undergo inward motions in a rotational manner. The transmission interface collapses the space in the central cavity, and TMDs move upwards to push and trigger substrate exit through the transporter translocation path.

We used all relevant ABCG2 cryo-EM coordinates to generate a refined structural model and to identify the organization of functional domains (Figure 1A). A vertical cross-section of homodimeric ABCG2 revealed two distinct cavities, a central cavity occupying the major space in the transmembrane domains, and a smaller upper cavity at the extracellular membrane region (Figure 1B). The cavities are separated by three gate-like motifs, the “entry gate” at the inner membrane leaflet, the “valve gate” resides between two cavities, and the “exit gate” which formed by ECL3 (Figure 1B). In the inward-facing (IF) state, the NBD dimer is open, maintaining a wider space in the intracellular region. The entry gate at the inner membrane leaflet holds an open mode, allowing for ligand access into the transport pore. The central cavity is enlarged, while the upper cavity is in a closed architecture (Figure 1C, left). ATP binding induces NBD dimerization that triggers the conformational switch from IF to outward-facing (OF) state that collapses the central cavity completely and pushes drugs toward the valve gate, while expanding the upper cavity thus creating an open exit gate, allowing for substrate release (Figure 1C, right). The movement illustrates how internal cavities and binding pockets may build the translocation pathway (Figure 1C). Of note, the distinct translocation channel in the OF state must originate from dynamic rearrangements in the core. We used PyMOL to analyze movements of individual residues during the conformational IF-OF switch, revealing high dynamics along the entire transporter core. NBD closure occurs in a rotational manner, while most residues around the TMD moved toward the center, thus collapsing the central cavity resulting in an upward-pushing to the upper cavity with an open gate to the extracellular region (Figures 1D and S12).

### Molecular docking reveals drug-binding pockets within the translocation channel

The drug binding regions within ABCG2 are enigmatic. To identify substrate-binding sites in ABCG2 variants, we conducted molecular blind docking using the CB-Dock (*cavity-detection guided blind docking*) tool.^39^^,^^40^ To verify that CB-Dock is a suitable, we docked mitoxantrone into an ABCG2 structure (SN38-bound ABCG2, 6VXJ), and aligned the output with related cryo-EM structures (mitoxantrone-bound ABCG2, 6VXI). The result indicated an identical binding position of mitoxantrone in the central cavity (Figure S13), confirming that the CB-Dock approach is suitable for binding site identification. Moreover, we used *MolProbity* to validate all models.^41^^,^^42^ Although all cryo-EM structures yielded solid quality, the PDB models 6VXJ, 6VXI, 6HZM, and 6HBU showed an optimal 100^th^ percentile of all-atom contacts and a strong *MolProbity* score. We used the 6VXJ and 6VXI coordinates as templates for the IF conformations, while 6HZM and 6HBU were used for OF states. The reliability of the homology models based on PDB (6VXJ) showed a good criteria cutoff at percentile ≥70^th^ with an acceptable *MolProbity* score (range around percentile 49^th^–58^th^) (Table S1).

ABCG2 exhibits broad multidrug specificity for both hydrophobic and hydrophilic compounds. To verify an impact on drug selectivity, we performed docking studies of ABCG2 variants (wild type [WT], R482G, K86M, and F431-F432) using four distinct substrates and the specific inhibitor. Mitoxantrone and pheophorbide A were used to look for binding pockets in WT, while daunorubicin and rhodamine 123 were employed as preferred substrates for the R482G mutant. Ko143, a selective high-affinity inhibitor of ABCG2, was used to elucidate inhibitor interactions. The ATPase-dead mutant, K86M, served as a non-functional control.

**Figure 2:**
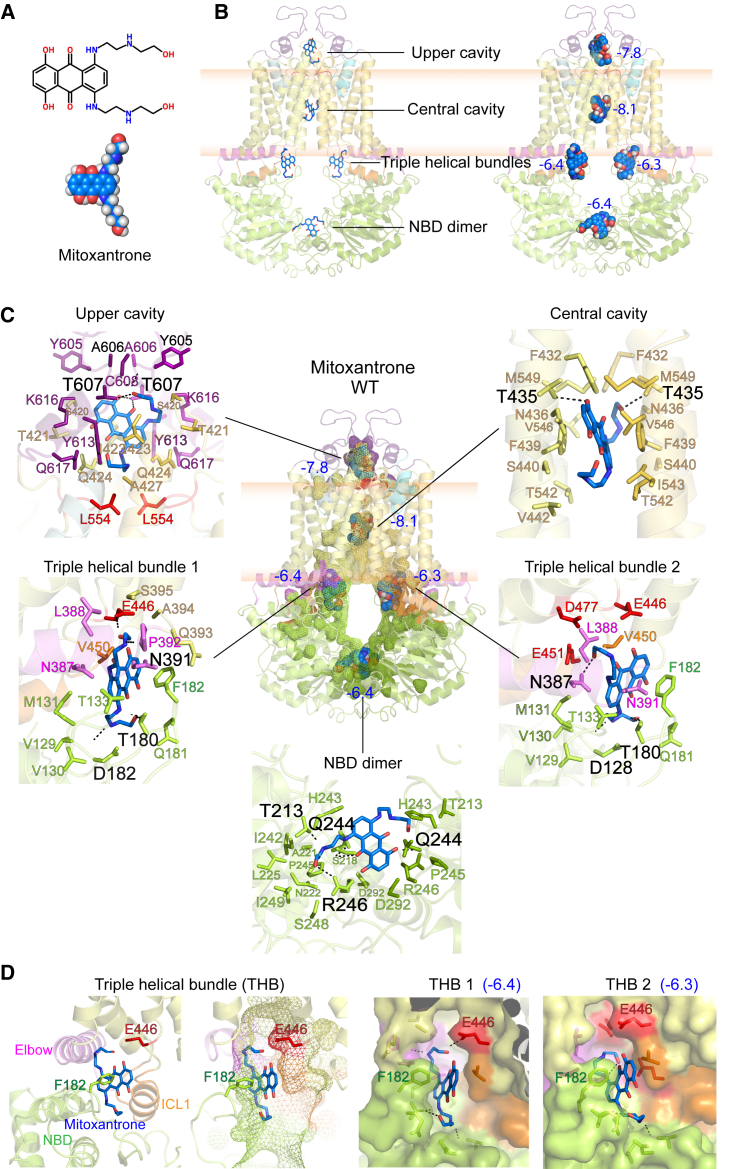
Modeling and molecular docking identifies five potential drug binding pockets in the ABCG2 translocation pathway (A) Chemical structure of mitoxantrone in stick and space-filling representations. (B) ABCG2 in an inward-facing state (6VXJ) reveals five potential binding pockets for mitoxantrone (blue stick or space-filling, left or right panels, respectively) with their binding affinity scores. (C) Potential binding pockets in the internal cavities of ABCG2 with possible interacting residues and binding affinity scores. Side-chains residues forming polar H-bond interactions are labeled in black, while non-polar hydrophobic interactions are indicated as color coded according to previous figures. Three essential negatively charged residues at the triple helical bundle are colored in red. (D) The ABCG2 substrate, mitoxantrone, is bound in the binding pocket close to the entry gate at triple helical bundle around transmission interface region. E446 (red) and F182 (green) provide side chains at the front of the entry gate.

In the IF state, ABCG2 reveals five potential binding regions for all substrates, including mitoxantrone (Figures 2 and S3), pheophorbide A (Figure S4), daunorubicin (Figure S5), rhodamine 123 (Figure S6), and Ko143 (Figure S7). Most substrates occupied the same binding pockets, suggesting a common translocation pathway in ABCG2. Mitoxantrone showed the optimal binding in the central cavity (binding score −8.1) and in the upper cavity (−7.8) (Figure 2B). Additionally, two binding sites were identified near the inner membrane leaflet, exactly at the highly conserved THB cluster (−6.4 and −6.3), suggesting the presence of an entry gate for drug recognition before access into the central cavity. We further analyzed the interacting residues at each predicted binding site using PyMOL and LigPlot+. Mitoxantrone exhibited both non-polar hydrophobic and polar hydrogen-bond interactions with multiple residues, highlighting their contribution to drug movement (Figure 2C). The interacting residues and bonds in the central cavity and upper cavity are summarized in Figure 4 and Table S2, respectively. Strikingly, R482G change distorted shape and flexibility around the THB, thus altering the recognition zone, and debilitating binding at the THB of the transmission interface (Figure S3D).

### E446 is essential for the entry gate and required for drug recognition

The conserved THB is present in all ABCG family members and essential for their function.^17^ We proposed that an entry gate resides at the inner bilayer leaflet. The conformational switch from IF-to-OF states may thus induce dynamic changes in this gate-like structure (Figure 1C), implying a mechanism for regulating drug access even before recognition by the primary binding region. Mitoxantrone docking in the binding pocket at the THBs revealed several hydrophobic interactions, including those in the NBD (V129, V130, M131, T133, Q181, and F182), the elbow helix (N389, L388, N391, and P392), and ICL1 (Q393, A394, S395, E446, V450, E451, and D477). Additionally, three polar interactions were observed with D128, T180, and N387 (Figure 2C). These putative binding pockets at the THBs were also observed for pheophorbide A (Figure S2C). A detailed analysis at both THBs identified E446 and F182 within the binding pockets, suggesting their involvement in drug recognition (Figure 2D). Substitutions at F182 with alanine, tyrosine, or tryptophan resulted in similar biochemical activities compared to WT (Figures S8D–S8M), indicating that the aromatic ring at F182 is not essential for ABCG2 function. By contrast, glutamate at E446 is required for ABCG2 function since it control drug access at the entry gate.^43^

### The central cavity forms a flexible binding pocket for diverse ligands

**Figure 3:**
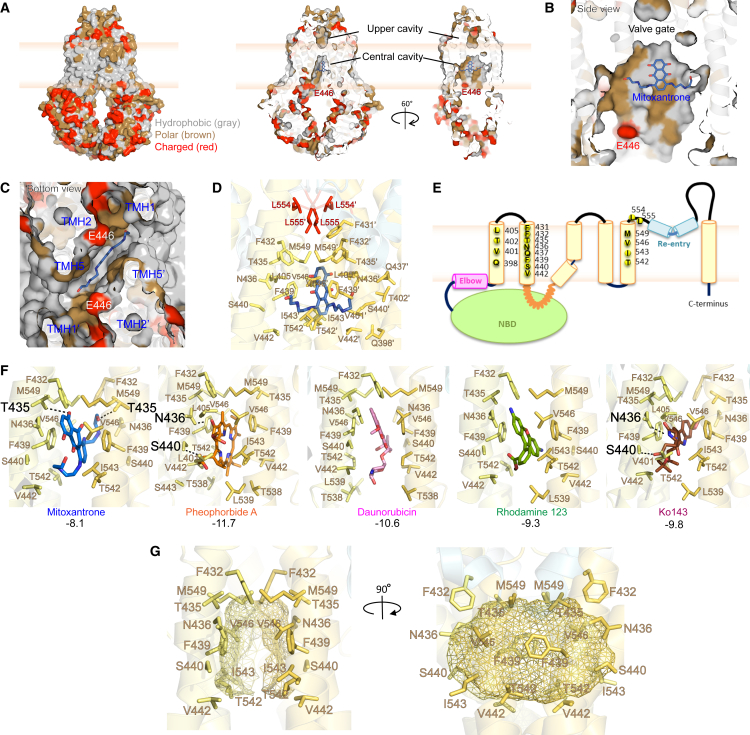
The central cavity of ABCG2 holds a major central binding pocket for drugs (A) Side-surface (left) and cross-section (right) views of ABCG2 structures (6VXI) are colored based on hydrophobic properties of side chains; hydrophobic (gray), polar (brown), and charged (red). ABCG2 holds most hydrophobic residues close to or as part of transmembrane-facing regions, and along the internal surface of the central cavity. Water-exposed regions contain several charged residues. (B) Zoom-in side-view of the central ABCG2 cavity show that the central cavity of ABCG2 is surrounded by hydrophobic-polar residues from TMH1-TMH2-TMH5. Residue E446, which is at the front of the entry gate, is the only charged residue in close proximity to the central cavity. (C) Zoom-in bottom-view of the central cavity. (D) The central cavity with relevant residues surrounding mitoxantrone at 6 Å-resolution. Side chains from TMH1-TMH2-TMH5 that are within 6 Å from mitoxantrone are indicated in yellow. (E) Membrane topology of an ABCG2 half transporter showing the N-terminal NBD (green), elbow helix (pink), six transmembrane helices (yellow), first intracellular loop (orange spring loop), re-entry helix (light blue), and the large extracellular loop 3. Interacting residues around the central binding cavity at 6 Å in TMH1, TMH2, and TMH5 (with the valve) are indicated. (F) Zoom-in side views of drug ligands docking in the central cavity of ABCG2. Side-chains of all interacting residues are depicted as stick representations using PyMOL. The side chain residues forming polar (H-bond) interactions are labeled in black, while non-polar (hydrophobic) interactions are indicated as color codes as in previous figures. The affinity binding scores are indicated below in black. (G) Common interacting residues in the ABCG2 central cavity are highlighted, with the side chains around the central cavity (yellow wireframe representation).

The central cavity of ABCG2 is the primary domain that accommodates distinct drugs as well as inhibitors (Figure 1C). Hydrophobicity analysis supported that lipid-exposed regions at the TMHs are highly hydrophobic, whereas water-exposed regions contain mainly charged residues (Figure 3A). Both cavities are surrounded by hydrophobic and polar residues, with E446 as the only negatively charged residue located just below the central cavity (Figures 3A–3C). The central cavity excludes other relevant charged residues, including R482, E451, or D477. We thus evaluated the lipophilicity of ligands by examining the *LogP* values. The results suggested that most ABCG2 substrates and inhibitors are highly lipophilic (Figure S1A), correlating with the hydrophobicity in both cavities (Figure 3B). We used PyMOL to spot all residues within a 6 Å proximity to mitoxantrone found in the mitoxantrone-bound cryo-EM model 6VXI (Figure 3D). The shape and size of central cavity were restricted by side chains from residues in 3 transmembrane helices, including TMH1 (Q398, V401, T402, and L405), TMH2 (F431, F432, T435, N436, Q437, F439, S440, and V442), and TMH5 (T542, I543, V546, and M549), respectively (Figure 3E).

**Figure 4:**
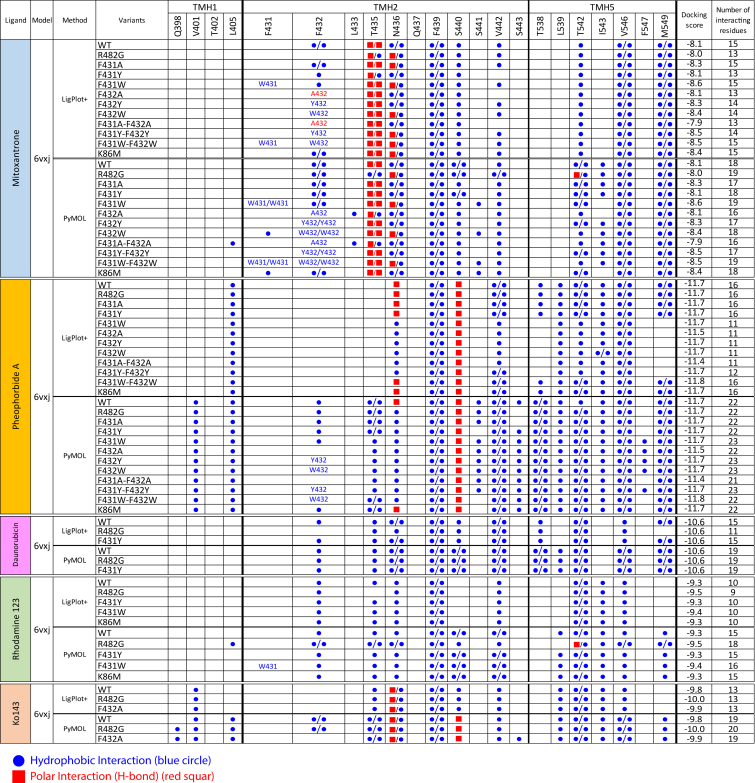
Summary of interacting residues lining the central cavity of ABCG2 Docking of ABCG2 variants with drug substrates and inhibitors at the central cavity was analyzed using the CB-Dock software tool to identify the interacting residues including arbitrary binding affinity scores. Interacting residues were visualized by PyMOL and LigPlot+. Hydrophobic (non-polar) interactions are shown in blue circles, while the hydrogen-bond interactions (polar) are indicated in red squares. Each symbol represents one interaction.

All key interacting residues in the central cavity from docking were identified by LigPlot+ and PyMOL (Figure 4). Each ligand shared overlapping interacting residues engaging both non-polar and polar contacts (Figure 3F). Mitoxantrone bound to the central cavity with the binding score of −8.1, interacting with 15–18 residues, including two H-bonds with T435 and hydrophobic interactions with F432, N436, F439, S440, V442, T542, I543, V546, and M439 (Figure 3F, first panel and Figure 4). Similarly, the ABCG2-specific pheophorbide A exhibited a stronger affinity of −11.7, engaging 16–22 residues, including hydrogen bonds with N436 and S440 and hydrophobic interactions from TMH1 (V401 and L405), TMH2 (F432, T435, N436, F439, S441, V442, and S443), and TMH5 (T538, L539, T542, I543, V546, and M549) (Figure 3F, second panel and Figure 4). Daunorubicin and rhodamine 123, alternative substrates of the R482G variant, also fit well into the central cavity with binding scores of −10.6 and −9.3, respectively, albeit with no polar interactions (Figure 3F, third and fourth panels, and Figure 4). The ABCG2-specific inhibitor Ko143 fit the central cavity with a score of −9.8, interacting with 13–19 residues, including two polar interactions (N436 and S440) and non-polar interactions engaging residues from TMH1 (Q398, V401, and L405), TMH2 (F432, T435, N436, F439, S440, and V442), and TMH5 (L539, T542, I543, V546, and M549), respectively (Figure 3F, fifth panel and Figure 4). Overall, all substrates shared ten common interacting residues in the central cavity, provided by TMH2 (F432, T435, N436, F439, S440, and V442) and TMH5 (T542, I543, V546, and M549). TMH2 and TMH5 are structural elements whose interactions shape the architecture of the central binding region (Figure 3G). Finally, multiple alignments using ClustalX2 demonstrated the evolutionary conservation of key residues among eukaryotic type II exporters, including ABCGs and yeast PDRs (Figure S9). While interacting residues from TMH1 and TMH5 exhibited a low conservation, TMH2 residues were highly conserved, especially all three phenylalanines (F431-F432-F439) in the core of the translocation pore. Importantly, the aromatic side chains of all phenylalanines face substrates trapped in the central cavity, thus confirming their critical role in ABCG2 transport function (Figure 7D).

### Drug efflux requires three phenylalanine pairs forming a “clamp-push-seal aromatic arm”

**Figure 5:**
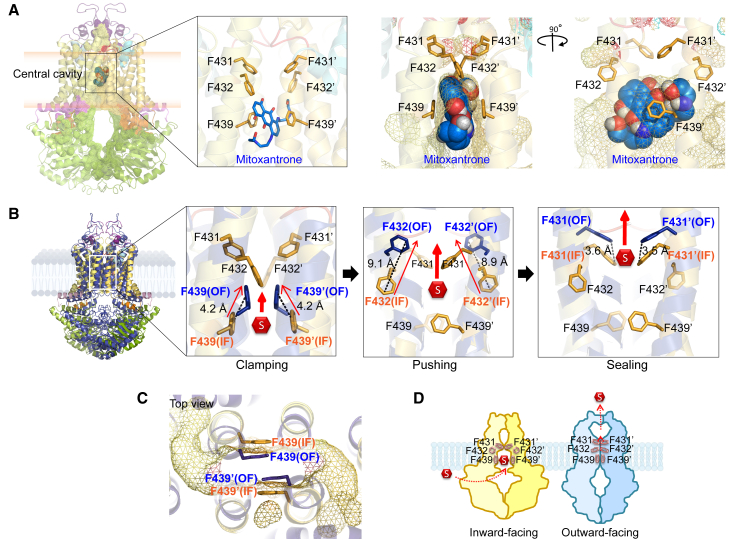
The paired phenylalanines, F439-F432-F431, form a “clamp-push-seal aromatic arm” to drive drug extrusion (A) Three pairing phenylalanines, F431-F432-F439, surround a mitoxantrone molecule (blue space-filling) in the central cavity. F439 and F432 provide hydrophobic interactions with mitoxantrone. (B) Three pairing phenylalanines drive drug efflux from the central cavity by acting as dynamic “clamp-push-seal aromatic arm” during the switch from IF (orange) to OF (blue) states. (C) Zoom-in top view in the central cavity indicates stacking of aromatic rings side chains of F439 in the IF (orange) versus OF states (blue) during the conformational switch of ABCG2. (D) The cartoon represents three series of actions by phenylalanines F439-F432-F431 in central cavity.

Three phenylalanines (F431-F432-F439) are highly conserved across ABCG-type transporters and provide their aromatic side chains surround ligand in the central cavity (Figures 5A and 7D), suggesting a crucial role, which we tested by extensive mutagenesis. Specifically, the F439 pair is centrally located and arranges the parallel aromatic rings in a hydrophobic stacking function that stabilizes ligands within the central cavity (Figure 5A).^44^ Importantly, this interaction is imperative for all ligands (Figure 4). During the IF-to-OF conformational switch, the aromatic side chains of F439 moved by about 4.2 Å, compressing the central cavity and facilitating ligand transfer to the F432 pair (Figures 5B and 5C). Thus, the aromatic side chain of the F439 pair form a “clamping arm” to trap ligands in the central binding region preceding substrate selection.

**Figure 6:**
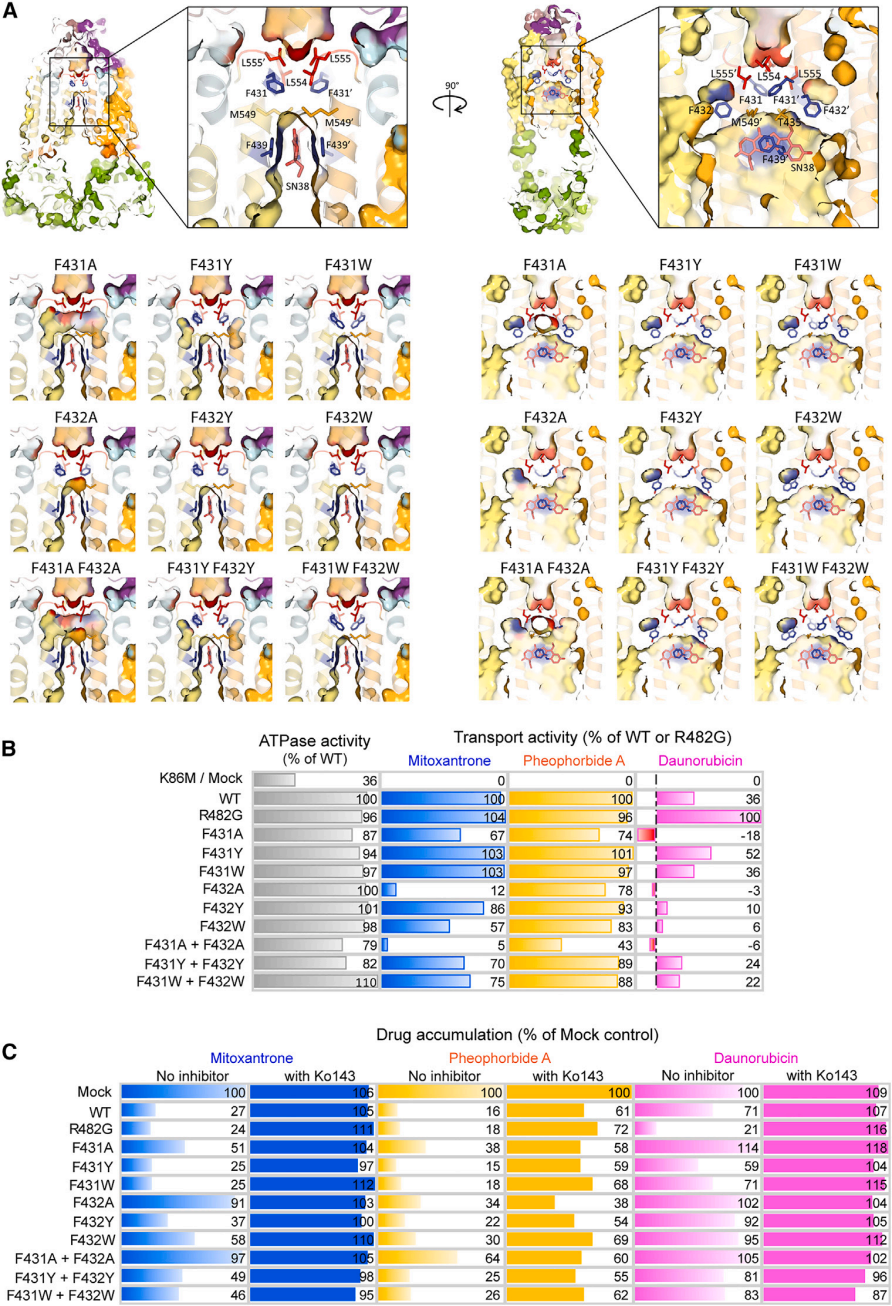
Three phenylalanine pairs in ABCG2 F439-F432-F431 are pivotal for drug efflux (A) Zoom-in cross-section structures of ABCG2 in the inward-facing state (PBD ID: 6VXJ), with two orientations of three phenylalanine side chains (blue) and side chains at the valve architecture. SN38 is in pink. At the valve, F431 side-chains are placed to support and seal the intact valve gate. (B) ATPase activity (gray) and drug efflux activities of ABCG2 variants with distinct drug substrates; mitoxantrone (blue), pheophorbide A (yellow), and daunorubicin (pink) were performed in HEK293 cell transfected with ABCG2 variants. Data were normalized from expressing cells and represented as percentage of WT (ATPase activity, mitoxantrone and pheophorbide A efflux) or R482G (daunorubicin efflux) as controls (*n* = 4–20). (C) Intracellular drug accumulations of mitoxantrone (blue), pheophorbide A (yellow), and daunorubicin (pink), in HEK293 cells expressing ABCG2 variants in the absence (gradient fill) and presence (solid fill) of ABCG2-specific inhibitor (Ko143). Color bars represented relative percentage of accumulation to mock control. All data point from 4 to 20 independent biological replicates and significant values are demonstrated in Figures S10D–S10G.

F432 forms the second pair of conserved phenylalanine residues above F439, N436, and T435. The aromatic side chain of F432 exhibited a long-range upward movement of approximately 9 Å during the IF-to-OF transition. This movement likely constitutes a “pushing action” to drive ligand translocation toward the upper cavity (Figure 5B). We generated homology models for all ABCG2 F431-F432 mutant variants using the cryo-EM 6VXJ as a template (Figure 6A). Interestingly, docking (Figure 4) demonstrated that all substrates required at least one hydrophobic interaction with residue F432. The F432A mutation dramatically disrupted the shape of the valve and expanded the central cavity (Figure 6A). Biochemical assays demonstrated that all F432 variants showed normal membrane localization, although F432Y showed moderately reduced expression levels to approximately 40% of WT (Figure S10). Drug efflux activities were calculated as percentage relative to WT or R482G. The negative mock control did not show transport activity for any substrate, whereas WT and R482G maintained transport for mitoxantrone and pheophorbide A with full ATPase activities. Daunorubicin was efficiently transported by the R482G variant (Figure 6B). Notably, F432A and F431A-F432A completely abrogated mitoxantrone efflux. This defect was significantly restored by tyrosine substitution (F432Y), while the bulky aromatic side chain in tryptophan of F432W retained approximately 60% function (Figure 6B). Thus, ABCG2-mediated drug efflux requires an aromatic side chain at position 432. Transport assays with pheophorbide A, a plant-derived chlorophyll metabolite with a *LogP* value of 4.45–4.24 (Figure S1), indicated that the alanine substitution around the binding region still allowed for efflux of extremely hydrophobic substrates such as pheophorbide A. Remarkably, all F432 variants showed only minor effect on pheophorbide A efflux (Figure 6B). Ko143 inhibition was slightly reduced in F432A and F432Y when compared to WT (Figure 6C). All F432 variants lacked daunorubicin efflux. Therefore, F432 does not impact ABCG2 substrate specificity (Figures 6B and 6C). The results confirmed that F432 establishes hydrophobic interactions with various ligands. The upward movement during the IF-OF switch activates a “pushing arm” to trigger drug translocation out of the central cavity (Figure 5D).

While F439 and F432 are only conserved in the first half of full PDR transporters, F431 is conserved in both half molecules (Figure S9). F431 supports the valve architecture, by using its side chain to build and act as a seal for the valve gate (Figures 5A and 6A). F431 showed a restricted motion by only about 3.5 Å during the IF-OF switch (Figure 5B). Docking data suggested that F431 may not directly interact with substrates in the central cavity (Figure 4) but plays a pivotal role by stabilizing the seal of the valve gate. Indeed, F431Y and F431W did not affect valve function (Figure 6A), whereas F431A disrupted its architecture, impairing efflux due to the loss of aromatic side chains. Functional analysis illustrated that F431A reduced protein expression to approximately 40% of WT, while F431Y and F431W showed minimal effects (Figures S10A and S10B). The F431 variants showed normal membrane localization (Figure S10C). The F431A decreased mitoxantrone and pheophorbide A efflux by about 70%, while F431Y and F432W retained full transport activity (Figure 6B). Thus, F431 is required for transport, likely by contributing to a functional valve structure, perhaps by establishing a sealing arm at the valve gate (Figure 6A).

To verify whether the neighboring residues F431 and F432 can compensate for each other, we generated double mutant variants (F431A-F432A, F431Y-F432Y, and F431W-F432W). All double mutations significantly decreased mature ABCG2 expression (Figure S10). The F431A-F432A totally impaired the valve structure and enlarged the central cavity (Figure 6A), leading to complete abrogation of mitoxantrone transport (Figure 6B), and diminished pheophorbide A efflux to about 40% of WT (Figure 6B). The F431Y-F432Y and F431W-F432W double mutants showed slightly reduced mitoxantrone efflux and no effect on pheophorbide A transport. None of the double mutants altered drug specificity (Figure 6B), and Ko143 led to full inhibition (Figure 6C). Overall, these data highlight the essential role of two aromatic rings at position 431 and 432 for establishing normal drug binding and subsequent ABCG2-mediated substrate efflux.

### Drug substrates engage in unique interactions with T435 and N436 in the central cavity

**Figure 7:**
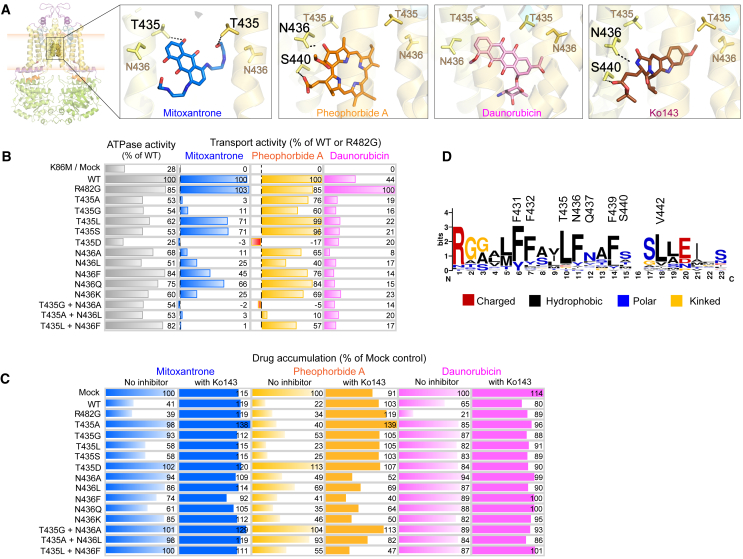
T435 and N436 interactions are unique and specific for each ligand (A) Zoom-in interactions of drug ligands with T435 and N436 in central cavity. Dotted lines indicated H-bond polar interactions. (B) ATP hydrolysis activity (gray) and drug efflux activity of ABCG2 variants with mitoxantrone (blue), pheophorbide A (yellow), and daunorubicin (pink) were done in HEK293 cell transfected with ABCG2 variants and measured. Results were normalized from expressing cells and represented as percentage of WT (ATPase activity, mitoxantrone, and pheophorbide A efflux) or R482G (daunorubicin efflux) with 4–20 independent experiments. (C) Drug accumulation levels of mitoxantrone (blue), pheophorbide A (yellow), and daunorubicin (pink), in HEK293 cells expressing ABCG2 variants in the absence (gradient fill) and presence (solid fill) of ABCG2-specific inhibitor (Ko143). Data are represented as color bars with relative percentage to mock control. Individual data point with statistic significant values is represented in Figures S11D–S11G. (D) Logo-plot representing relative frequencies of each residue in TMH2 of mammalian ABCG2 versus the first half of fungal PDRs. Interacting residues of human ABCG2 were labeled above the plot. Properties of the residues are indicated as color codes; charged (red), hydrophobic (black), polar (blue), and kinked (yellow).

The mechanisms underlying the extreme broad substrate selectivity of ABCG2 remain enigmatic. While mammalian ABCGs and yeast PDRs hold a conserved leucine and phenylalanine at position 435–436, human ABCG2 uniquely contains a threonine-asparagine pair instead (Figure 7D). T435 and N436 reside above F439, and docking data suggested that T435 and N436 must engage with most substrates (Figure 4). Remarkably, distinct interactions for both T435 and N436 were observed (Figure 7A), and we thus hypothesized that T435-N436 may be critical for classifying ligands during their stabilization at the F439 “clamping arm.” Thus, T435-N436 represent key residues that distinguish the multidrug ABCG2 exporter from other type II exporters.

We generated 13 ABCG2 variants by substituting T435 and N436 with various amino acids to obtain the T435A, T435G, T435L, T435S, T435D, N436A, N436L, N436F, N436Q, and N436K mutants. While double mutation variants were created based on conserved residues present in the cholesterol lipid half transporter ABCG5 (T435G-N436A), ABCG8 (T435A-N436L), as well as ABCG1, ABCG4, and yeast PDRs (T435L-N436F). Most T543-N436 variants maintained nearly normal protein expression levels, with moderate reductions observed in some mutants (T435L, N436Q, N436K, and double mutant T435L-N436F). Only the T435D mutant completely abrogated mature protein expression levels and fully abrogated transport for all substrates (Figures 7B and S11). The docking results indicated that mitoxantrone requires two polar interactions with T435 (Figure 7A). Consequently, the absence of polar side chains in T435A and T435G totally abrogated mitoxantrone efflux, while transport was restored in T435L and T435S. N436 engages two hydrophobic interactions with mitoxantrone (Figure 7A), also involved in drug transport. Only N436Q retained function, whereas all other mutants (N436A, N436L, N436F, and N436K) showed reduced activities (Figure 7B). Notably, all double mutants (T435G-N436A, T435A-N436L, and T435L-N436F) completely disrupted mitoxantrone efflux, strongly suggesting that T435 facilitates polar interactions with substrates, while N436 engages in hydrophobic interactions, all of which are necessary for mitoxantrone efflux (Figure 4). Docking of pheophorbide A revealed one hydrogen bond with N436 and at least one hydrophobic interaction with T435, and one H-bond with S440 (Figures 4 and 7A). Most single mutations at T435 or N436 maintained transport function, with moderate reductions in T435G and N436L. However, T435G-N436A and T435A-N436L almost fully abrogated pheophorbide A efflux, suggesting that at least one interaction provided by either T435 or N436 is necessary and sufficient for pheophorbide A efflux (Figure 7B). Surprisingly, Ko143 inhibition were reduced in all N436 variants (Figure 7C), and docking results suggested that Ko143 requires one polar and one hydrophobic interaction with N436 side chains from both ABCG2 halves (Figure 7A). Therefore, mutations at N436 may impair the binding of Ko143, while maintaining pheophorbide A efflux, since extrusion of this compound requires only one interaction with either T435 or N436.

The molecular docking suggested that R482G substrates such as daunorubicin and rhodamine 123 did not engage in polar interactions within the central cavity but instead relied on high lipophilicity for non-polar interactions, especially with the residues T435 and N436 (Figure 4). Moreover, T435-N436 mutants did not alter drug specificity. Therefore, these results strongly indicated that T435 and N436 act as key substrate selector, enabling the recognition of both substrates and inhibitors within the central cavity.

### ECL3 operates as an exit gate to regulate drug release from the upper cavity

**Figure 8:**
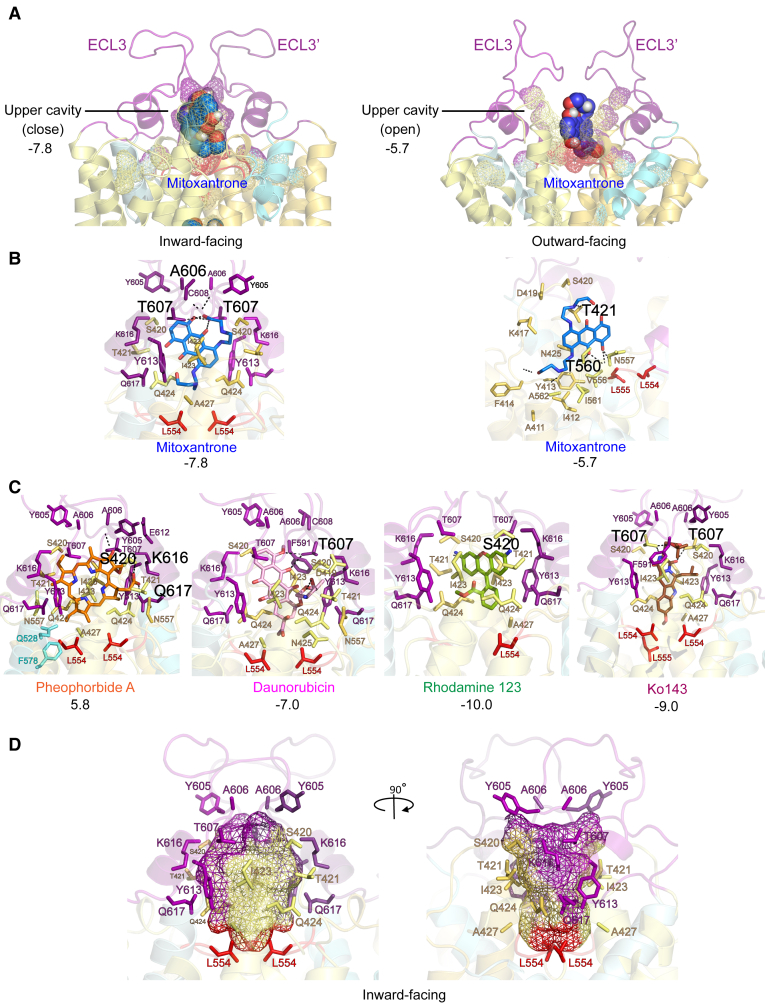
ECL3 constitutes an exit gate for drug release from the upper cavity (A) Structure of ABCG2 at the extracellular roof is depicted in both conformations in cartoon ribbons mode; extracellular loop 3 (ECL3, purple), re-entry helix (cyan), transmembrane helices (yellow), mitoxantrone (blue space-filling) with binding scores obtained from docking. Structural dynamics and transitions of ECL3 regulate shape and release from the upper cavity. (B) Zoom-in of mitoxantrone (blue stick) in the upper cavity of ABCG2 transporter in an IF (left) and an OF (right) states with their affinity binding scores. All interacting residues identified by PyMOL are depicted with side chains in stick representations. Polar (H-bond) interactions are labeled in black with black dotted lines. Apolar hydrophobic interacting residues are colored according to their motifs shown in panel. (C) Zoom-in of drug ligands in stick representations; pheophorbide A (orange), daunorubicin (pink), rhodamine 123 (green), and Ko143 (brown), in the upper cavity of ABCG2 in an inward-facing state. Hydrogen-bond interactions are demonstrated in black, while hydrophobic interactions are labeled as color codes. The binding scores are indicated below. (D) Common interacting residues in the ABCG2 at an inward-facing state are highlighted as colored side chains and color wireframe representation around the upper cavity.

Several motifs such as the re-entry helix, the hydrophobic di-leucine valve, and the connected extracellular loops (ECLs) contribute to the roof architecture, including stabilizing disulfide bonds and salt bridges.^18^ ECL3 plays a vital role following the IF-to-OF switch, acting as a terminal gate that controls shape and size of the upper cavity that allows for drug extrusion (Figure 8A). In the IF state, the upper cavity is almost fully sealed, owing to the di-leucine valve that ensures a tight separation from the central cavity, perhaps to prevent re-entering of substrates into the translocation pore. This is necessary to ensure unidirectional transport.^18^

Docking results revealed potential binding regions in the upper cavity, since mitoxantrone bound with an affinity score of −7.8, engaging three possible hydrogen bonds (two with T607 and one with Y613), along with additional hydrophobic interactions with several ECL1 residues (S420, T421, I423, Q424, A427, L554, Y605, A606, Y613, and K616) (Figure 8B, left). By contrast, the OF state holds an enlarged upper cavity, where all interactions with ECL3 disappear (Figure 8B, right). This movement also reduces the binding affinity of mitoxantrone to −5.7, which is necessary for substrate release (Figure 8A). Several drugs exhibited distinct affinities within the upper cavity, correlating with their structures and chemical space, yielding distinct binding scores for mitoxantrone (−7.8) (Figure 8A), pheophorbide A (+5.8), daunorubicin (−7.0), rhodamine 123 (−10.0), and Ko143 (−9.0) (Figure 8C). Notably, the large space of pheophorbide A did not fit well into the upper cavity in the IF state, thus explaining the positive affinity score. The potential interacting residues identified in the upper cavity are summarized in Table S2. The results also revealed common interacting residues of several substrates in the roof architecture, including ECL1 (S420, T421, I423, Q424, and A427), the di-leucine valve (L554) as well as ECL3 (Y605, A606, T607, Y613, K616, and Q617) (Figure 8D; Table S2). Taken together, the exit gate mainly exploits dynamic structural rearrangements dictated by ECL3 to control the opening of the upper cavity for substrate release to the extracellular space.

### Proposed molecular transport mechanism along the translocation path

**Figure 9:**
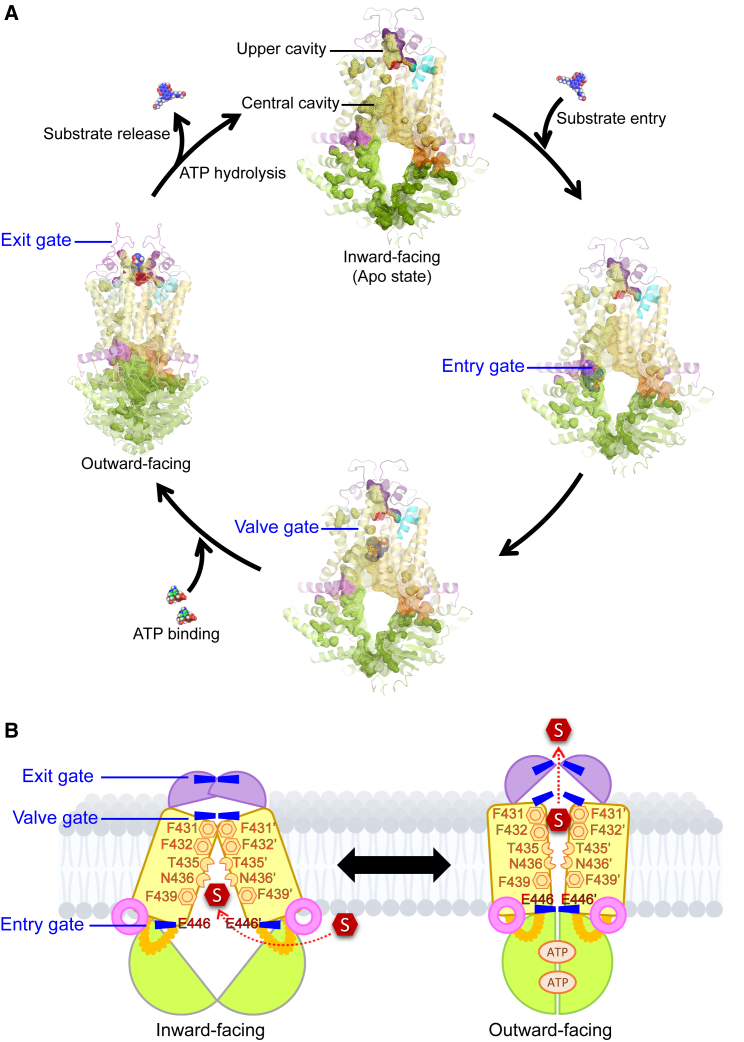
ABCG2 integrates ligand recognition with conformational switches during drug efflux (A) Transport cycle of ABCG2 engages three regulatory gates formed along translocation channel. (B) Model of drug recognition and extrusion mechanisms during the transport cycle in a triple-gate ABCG2 transporter. The cartoons represent ABCG2 in two conformations of the drug translocation path in the catalytic cycle. NBD (green), elbow helix (pink), ICL1 (orange), TMD (yellow), ECL3 (purple), tree gates (blue), and substrate (red hexagon).

The ABCG2 drug efflux transporter operates as a peristaltic pump, exporting a wide range of xenobiotic substances.^18^ The present data suggested a potential translocation pore within ABCG2 as the primary pathway for unidirectional substrate efflux (Figure 9). In the apo drug-free state, ABCG2 adopts an IF conformation with two distinct cavities, offering accessible binding pockets for substrates. The highly conserved THB at the transmission interface provides an “entry gate” with a potential binding region holding the essential residue E446, which plays a vital role in drug recognition before access into the central cavity. The central cavity offers a spacious binding pocket for various substrates and inhibitors and orchestrates drug movement by utilizing three pairs of conserved phenylalanines (F439-F432-F431), as well as the selector residues T435 and N436. First, ligands are recognized and stabilized in the central cavity by hydrophobic interactions with two F439 rings acting as “clamping arms.” Specific interactions with two selector residues, T435 and N436, classify substrate and inhibitors. The binding of two ATP molecules initiates NBD dimer formation, thus closing the entry gate and creating a substrate-trapped closed pore. Consequently, the F439 pair compresses the central cavity, generating peristaltic pressure in the transport channel. The F432 pair establishes hydrophobic interactions with drugs during the IF-to-OF switch, and their wide-range movements drive a “pushing arm” motion for upwards movement toward the valve gate. Finally, the F431 pair contributes to the valve gate by strengthening the hydrophobic seal separating the central and upper cavities. ECL3 serves as an exit gate that regulates drug release, completing the transport cycle (Figure 9).

Taken together, this work identifies pivotal residues that form binding regions lining the drug translocation pore in the core of ABCG2. Our findings show that at least gates are required to control the efflux of multiple drugs through the central pore. The results provide an explanation for the promiscuously broad ligand selectivity, and offer mechanistic insights governing substrate movement during ABCG2-mediated multidrug efflux.

## Discussion

ABCG2 is pivotal for physiological detoxification across barrier tissues, but its ectopic expression levels also mediates MDR phenomena in cancer.^8^^,^^45^^,^^46^^,^^47^ Despite the availability of 25 cryo-EM ABCG2 structures, most mechanistic details regarding substrate selectivity, drug recognition, and gating mechanisms along translocation pore remain elusive. Here, we dissect in detail the dynamics of the putative ABCG2 substrate translocation channel by integrating structural analysis and computational approaches with experimental validation through biochemical assays to identify critical regulatory steps governing multidrug efflux through the translocation pathway. The structural modeling using available ABCG2 cryo-EM structures^16^^,^^28^^,^^29^^,^^30^^,^^31^^,^^32^^,^^33^^,^^34^^,^^35^ predicts a symmetric channel arrangement with two distinct cavities, separated by three apparent gates. The integrated structural models suggest that conformational flexibility allows for massive rearrangements during substrate efflux. This notion is strongly supported by mutagenesis and experimental validation of key predictions from the structures concerning the discrete gates involved in controlling drug extrusion. Interestingly, although the exporter type II proteins share similar folding patterns,^20^ the shape and size of the ABCG2 translocation path appears quite different from other ABCGs or the conserved fungal PDRs.^48^ This difference may be attributed to the fact that transporter employs a specific mechanism to feed the transport channel after drug recognition and substrate efflux. For example, differences in shape and size of internal cavities in yeast Pdr5 versus mammalian ABCGs may arise for two reasons. First, Pdr5 is a full transporter with an intrinsically asymmetric fold due to the non-identical nature of its first and second halves as confirmed by Pdr5 cryo-EM structures.^48^ Second, unlike ABCG2, the asymmetric Pdr5 multidrug exporter holds one degenerate NBD unable to hydrolyze ATP, yet still allows for a normal catalytic cycle.^49^^,^^50^ This structural asymmetry most likely affects the conformational dynamics of the translocation channel thus altering substrate interaction and selectivity.^48^^,^^50^^,^^51^

Blind docking can predict potential binding regions, thus disclosing possible access paths for substrates and/or inhibitors in the transporter core. This provides a reasonable explanation for the extremely broad ABCG2 substrate selectivity. The architecture around the highly conserved THB at the transmission interface establishes a potential binding zone referred to as the “entry gate.” Our previous studies showed that three negatively charged residues in this region, E446, E451, and D477, are important for ABCG2 function.^43^ In particular, the side chain of E446 is absolutely essential for drug efflux. Interestingly, E446 is not conserved in the type II exporter family, suggesting it plays a unique role in substrate recognition and access in ABCG2. Additionally, the glutamate residue of E219 in the heme-export pump HrtBA from Gram-positive bacteria is also essential for heme-binding and transport.^52^ The ABCG2 R482G variant may hold an even more flexible entry gate, since it expands the broad substrate spectrum to rhodamine compounds. However, we cannot rule out that the broad ABCG2 spectrum may engage more than one entry gate. For example, additional entry gate(s) for highly hydrophobic or highly hydrophilic substances may allow for access from the lipid bilayer or even from the cytoplasm in the IF state, with separated NBDs, respectively. In any case, the central cavity of ABCG2 provides an elastic spacious binding region showing strong binding affinities for many ligands. The large size of the central cavity enhances the peristaltic pushing action that triggers or accompanies the conformational IF-to-OF switch that is the key driver for drug extrusion.

Aromatic side chains play important roles in establishing gating mechanisms in many membrane transporters, including ABCB1,^53^^,^^54^ bacterial manganese importer (PsaBC),^55^ *Streptomyces peucetius* (DrrAB),^56^ RND transporters,^57^ as well as in the MCE protein family.^58^ Moreover, phenylalanines also form hydrophobic clusters in the core of ABCG1,^59^ as well as ABCG5/ABCG8.^60^ In ABCG2, substrates are pushed toward the pore containing the three conserved phenylalanines F431, F432, and F439. ABCG2 appears to recognize and trap drug substrates using by two parallel F439 aromatic side chains that face drugs within the pore, and act as “clamping arms.” The clamping of trapping contributes to the compression of the central cavity, thereby generating peristaltic pressure.^44^ The second pair from both F432 sustains hydrophobic interactions and supports significant movements during the conformational switch, thus serving as a “pushing arm” for unidirectional transport. Finally, the conserved F431 aromatic pair contributes to the formation of a regulated valve gate, although F431 variants have only a minor impact on function. Our docking data support that F431 plays a role in efflux by stabilizing the structure at the valve seal.

Our findings demonstrate that the ABCG2-specific residues T435 and N436 residing in the central cavity act as selectors to classify ligand interactions, which is fully consistent with a recent independent report that identifies N436 as a substrate and inhibitor discriminator.^61^ In contrast, other type II ABC exporters possess different residues such as leucine and phenylalanine at the equivalent position, suggesting that T435 and N436 play pivotal roles in determining specificity and selectivity of ABCG2. Indeed, our functional assays confirm that specific interactions with T435 and N436 are imperative and distinct for each ligand (Figure 4), strongly supporting their roles in drug recognition.

Finally, the upper cavity facing the outer lipid bilayer leaflet establishes the final domain of the translocation pore preceding drug release into the extracellular space. Notably, the upper cavity is completely sealed in the IF state, whereas the upper cavities in ABCG5/ABCG8, ABCG1 and Pdr5 appear more open with an already open lid.^19^^,^^62^ Our data confirm that the movement of ELC3 enlarges the upper cavity, thereby reducing substrate affinities to allow for drug release through the dynamically opening roof.

While we provide a validated and feasible model for ABCG2-mediated multidrug efflux, we cannot exclude alternative mechanisms, such as a Brownian ratchet. Notably, a Brownian ratchet mechanism may apply for ABC transporters based on theoretical calculations,^63^ but no experimental evidence is currently unavailable. We believe that substrate recognition and dynamics may also be influenced by other possible factors that impact the conformational changes of membrane proteins in physiological conditions to ensure the unidirectional nature of transport.

In conclusion, we demonstrate that the human ABCG2 efflux pump engages a highly dynamic multistep translocation process, supported by the integrating data from comparing cryo-EM structures, homology modeling, computational docking, and genetic validation as well as functional biochemical validation of mutant variants. This process enables drug transport while allowing access at various entry points in the “breathing” pore. Key residues, such as E446, T435, and N436, play crucial roles in drug recognition, selection, and movement, while F439 and F432 pivotal for facilitating transport. Furthermore, the flexibility of ELC3 is crucial for maintaining a closed core and regulated drug release. Our findings suggest that specific residues may offer new therapeutic options, including the design of inhibitors that target regulatory gates to combat cancer MDR.

### Limitations of the study

While this study provides valuable insights into the molecular mechanisms underlying the function of the ABCG2 transporter, it is important to acknowledge a significant limitation. The computational resources available for this investigation were insufficient to facilitate molecular simulations encompassing the entire translocation pathway of the ABCG2 transporter. Conducting a comprehensive simulation of the complete translocation process necessitates substantial computational power and extended simulation times, which exceeded the scope of this study. Consequently, our simulations were confined to selected key steps within the translocation pathway, rather than capturing the full dynamic process. Future research employing high-performance computing platforms or more advanced simulation methodologies may offer a more exhaustive characterization of the translocation mechanism of the ABCG2 transporter.

## Resource availability

### Lead contact

Further information and requests for resources and reagents should be directed to and will be fulfilled by the lead contact, Professor Dr. Karl Kuchler (kuchlerkarl1@gmail.com).

### Materials availability

Further information and requests for resources and reagents should be directed to and will be fulfilled by the lead contact, Professor Dr. Karl Kuchler (kuchlerkarl1@gmail.com).

### Data and code availability

• All relevant data supporting the findings of this manuscript are available from the authors and/or included in the manuscript or supplemental information.
• Data are available as of the date of publication.
• This paper does not report original code.
• Any additional information required to reanalyze the data reported in this paper is available from the lead contact upon request.
• The raw datasets, including coordinates of structural models in this study, are available at https://github.com/kakulab/ABCG2-CellReports.git.

## Acknowledgments

These studies were supported by a grant from 10.13039/501100002428Austrian Science Fund to K.K. (FWF-SFB035). We thank Oliver Langer for providing ABCG2 substrates and inhibitors.

## Author contributions

N.K. and K.K. conceptualized this study. N.K. designed and performed all experiments and computational analysis. N.K. and K.K. interpreted the data and wrote the manuscript.

## Declaration of interests

The authors declare no competing interests.

## STAR★Methods

### Key resources table

**Table:** 

REAGENT or RESOURCE	SOURCE	IDENTIFIER
**Antibodies**		

Mouse monoclonal anti-ABCG2 (BXP-21)	Santa Cruz Biotechnology	Cat#sc-5822
Rabbit monoclonal anti-β actin (D6A8)	Cell Signaling Technology	Cat#8457S
Goat anti-mouse IgG (H+L) secondary antibody, IRDye 800CW	LI-COR	Cat#926-32210
Goat anti-rabbit IgG (H+L) secondary antibody, IRDye 680CW	LI-COR	Cat#926-68071

**Chemicals, peptides, and recombinant proteins**		

Dulbecco’s modified Eagle’s medium (DMEM) high glucose + glutamine	Thermo Fisher Scientific	Cat#41966-052
Fetal calf serum	Sigma-Aldrich	Cat#F7524
Bovine serum albumin	Sigma-Aldrich	Cat#A2153
Polyethylenimine, Linear (MW 25000)	Polyscience Europe GmbH	Cat#23966-2
G418	Santa Cruz Biotechnology	Cat#sc-29065
Mitoxantrone	Sigma-Aldrich	Cat#M6545
Pheophorbide A	Santa Cruz Biotechnology	Cat#sc-264070B
Rhodamine 123	Sigma-Aldrich	Cat#83702
Methotrexate	THP Medical Products Vertriebs GmbH	Cat#MCE-HY-14519
Daunorubicin HCl	Bio-Diagnostica Vertrieb GmbH	Cat#CBS-MD11679
Alpha-Naphthoflavone (or 7,8-benzoflavone)	TCI Deutschland GmbH	Cat#B0056
Ko143	Sigma-Aldrich	Cat#K2144
FK506 (Tacrolimus)	THP Medical Products Vertriebs GmbH	Cat#MCE-HY-13756
Flavopiridol	THP Medical Products Vertriebs GmbH	Cat#MCE-HY-10005
KS176	THP Medical Products Vertriebs GmbH	Cat#MCE-HY-19753
Sodium Orthovanadate	New England Biolabs GmbH	Cat#P0758S
7-Dehydrocholesterol	Santa Cruz Biotechnology	Cat#sc-214398
cOmplete™ Proteasehemmer-Cocktail	Sigma-Aldrich	Cat#4693116001
30% Acrylamide/Bis Solution, 37.5:1	Bio-Rad Laboratories	Cat#1610159
Page Ruler Prestained Plus Protein Ladder	Thermo Fisher Scientific	Cat#26619
DMSO cell culture treated	Sigma-Aldrich	Cat#D4540
Phusion HighFidelity DNA-Polymerase	Thermo Fisher Scientific	Cat#F530S
DpnI	New England Biolabs GmbH	Cat#R0176s
NotI (FastDigest)	Thermo Fisher Scientific	Cat#FD0594
Potassium antimony(III) tartrate hydrate	Sigma-Aldrich	Cat#244791-100G
Adenosine 5'-triphosphate disodium salt (ATP)	Sigma-Aldrich	Cat#A7699-1G
Ouabain octahydrate	Sigma-Aldrich	Cat#O3125-250MG
CHEMS (cholesteryl hemisuccinate), powder	Sigma-Aldrich	Cat#850524P
DNase I recombinant, Rnase-Free solution	Sigma-Aldrich	Cat#471672
Triton X-100	Sigma-Aldrich	Cat#X100
DAPI	Sigma-Aldrich	Cat#D9542
Hoechst 33342	Sigma-Aldrich	Cat#14533

**Experimental models: Cell lines**		

HEK293	ATCC	Cat#CRL-1573

**Oligonucleotides**		

See Table S3 for full oligonucleotide list	Eurofins Genomics GmbH	N/A

**Recombinant DNA**		

pcDNA3.1(-)-FLAG-ABCG2 (WT)	Khunweeraphong et al.^17^	N/A
pcDNA3.1(-)-FLAG-ABCG2-R482G	Khunweeraphong et al.^17^	N/A
pcDNA3.1(-)-FLAG-ABCG2-K86M	Khunweeraphong et al.^17^	N/A
pcDNA3.1(-)-FLAG-ABCG2-F182A	This study	N/A
pcDNA3.1(-)-FLAG-ABCG2-F182Y	This study	N/A
pcDNA3.1(-)-FLAG-ABCG2-F182W	This study	N/A
pcDNA3.1(-)-FLAG-ABCG2-F431A	This study	N/A
pcDNA3.1(-)-FLAG-ABCG2-F431Y	This study	N/A
pcDNA3.1(-)-FLAG-ABCG2-F431W	This study	N/A
pcDNA3.1(-)-FLAG-ABCG2-F432A	This study	N/A
pcDNA3.1(-)-FLAG-ABCG2-F432Y	This study	N/A
pcDNA3.1(-)-FLAG-ABCG2-F432W	This study	N/A
pcDNA3.1(-)-FLAG-ABCG2-F431A - F432A	This study	N/A
pcDNA3.1(-)-FLAG-ABCG2-F431Y - F432Y	This study	N/A
pcDNA3.1(-)-FLAG-ABCG2-F431W - F432W	This study	N/A
pcDNA3.1(-)-FLAG-ABCG2-T435A	This study	N/A
pcDNA3.1(-)-FLAG-ABCG2-T435G	This study	N/A
pcDNA3.1(-)-FLAG-ABCG2-T435L	This study	N/A
pcDNA3.1(-)-FLAG-ABCG2-T435S	This study	N/A
pcDNA3.1(-)-FLAG-ABCG2-T435D	This study	N/A
pcDNA3.1(-)-FLAG-ABCG2-N436A	This study	N/A
pcDNA3.1(-)-FLAG-ABCG2-N436L	This study	N/A
pcDNA3.1(-)-FLAG-ABCG2-N436F	This study	N/A
pcDNA3.1(-)-FLAG-ABCG2-N436Q	This study	N/A
pcDNA3.1(-)-FLAG-ABCG2-N436K	This study	N/A
pcDNA3.1(-)-FLAG-ABCG2-T435G - N436A	This study	N/A
pcDNA3.1(-)-FLAG-ABCG2-T435G - N436L	This study	N/A
pcDNA3.1(-)-FLAG-ABCG2-T435L - N436F	This study	N/A
pEGFPC1-GFP-ABCG2 (WT)	Khunweeraphong et al.^43^	N/A
pEGFPC1-GFP-ABCG2-R482G	Khunweeraphong et al.^43^	N/A
pEGFPC1-GFP-ABCG2-K86M	Khunweeraphong et al.^43^	N/A
pEGFPC1-GFP-ABCG2-F182A	This study	N/A
pEGFPC1-GFP-ABCG2-F182Y	This study	N/A
pEGFPC1-GFP-ABCG2-F182W	This study	N/A
pEGFPC1-GFP-ABCG2-F431A	This study	N/A
pEGFPC1-GFP-ABCG2-F431Y	This study	N/A
pEGFPC1-GFP-ABCG2-F431W	This study	N/A
pEGFPC1-GFP-ABCG2-F432A	This study	N/A
pEGFPC1-GFP-ABCG2-F432Y	This study	N/A
pEGFPC1-GFP-ABCG2-F432W	This study	N/A
pEGFPC1-GFP-ABCG2-F431A - F432A	This study	N/A
pEGFPC1-GFP-ABCG2-F431Y - F432Y	This study	N/A
pEGFPC1-GFP-ABCG2-F431W - F432W	This study	N/A
pEGFPC1-GFP-ABCG2-T435A	This study	N/A
pEGFPC1-GFP-ABCG2-T435G	This study	N/A
pEGFPC1-GFP-ABCG2-T435L	This study	N/A
pEGFPC1-GFP-ABCG2-T435S	This study	N/A
pEGFPC1-GFP-ABCG2-T435D	This study	N/A
pEGFPC1-GFP-ABCG2-N436A	This study	N/A
pEGFPC1-GFP-ABCG2-N436L	This study	N/A
pEGFPC1-GFP-ABCG2-N436F	This study	N/A
pEGFPC1-GFP-ABCG2-N436Q	This study	N/A
pEGFPC1-GFP-ABCG2-N436K	This study	N/A
pEGFPC1-GFP-ABCG2-T435G - N436A	This study	N/A
pEGFPC1-GFP-ABCG2-T435G - N436L	This study	N/A
pEGFPC1-GFP-ABCG2-T435L - N436F	This study	N/A

**Software and algorithms**		

PyMOL	The PyMOL Molecular Graphics System	https://pymol.org/
CB-Dock	Liu, Grimm et al.^39^	http://clab.labshare.cn/cb-dock/
LigPlot+	Laskowski and Swindells^64^	https://www.ebi.ac.uk/thornton-srv/software/LigPlus/
MolProbity	Davis, Leaver-Fay et al.^42^	http://molprobity.biochem.duke.edu/
GalaxyWEB	Ko, Park et al.^65^	https://galaxy.seoklab.org/
ALOGPS 2.1	Tetko and Poda^66^	https://vcclab.org/lab/alogps/
WebLogo: A sequence logo generator	Crooks, Hon et al.^67^	https://weblogo.berkeley.edu/logo.cgi
ZEN	ZEISS	https://www.zeiss.com
Image Studio	LI-COR	https://www.licor.com/bio/image-studio/
GraphPad Prism	GraphPad Software Inc.	N/A
FlowJo software	FlowJo Inc. (Standford University)	N/A

**Other**		

The raw datasets, including coordinates of structural models	This study	https://github.com/kakulab/ABCG2-CellReports.git

### Experimental model and study participant details

#### Cell culture

HEK293 cells were cultured in Dulbecco’s modified Eagle’s medium (DMEM) high glucose + glutamine, supplemented with 5% heat inactivated Fetal calf serum with humidity at at 37°C and 5% CO_2_. The transfection was performed using self-made poly-ethyleneimine (PEI). For stable cell lines, HEK293 cells were maintained in cultured medium supplemented with 0.9 g/L of G418. Cell lines utilized in this study were screened for Mycoplasma contamination, and not contamination was detected.

### Method details

#### Sequence analysis

Amino acid sequences were obtained from NCBI database. The accession numbers of protein sequences were indicated in the alignment result (Figure S9). BioEdit and ClustalX2 were used to generate multiple sequence alignments of ABCGs/PDRs proteins. The regions of TMH1, TMH2 and TMH5 (with Valve) were highlighted based on the corresponding regions in human ABCG2. The sequence Logos plot graphic was generated via WebLogo: A sequence logo generator^67^ to present the sequence conservation based on nucleic acid multiple sequence alignment. The overall height indicates the conservation of each position, while the height of each amino acid in the stack related to the frequency of the corresponding residue at that position. The colors of residues reflect their chemical properties, red: charged, black: hydrophobic, polar: blue, yellow kinked, respectively.

#### Structural homology modeling and refinement

The molecular structures were analyzed and visualized using PyMOL, v1.20. (Schrödinger, LLC, New York, NY, USA). The structural models of human ABCG2 variants were created based on the cryo-EM structures of human ABCG2 as templates (6VXJ, 6VXI for inward-facing conformations and 6HZM, 6HBU for outward-facing conformations).^28^^,^^30^ To create and provide starting coordinates for ABCG2 variants, allowing investigation of the impact on the ABCG2 structure, a series of point mutations were introduced using PyMOL. The generated structural models of all variants were validated by MolProbity^41^^,^^42^ to evaluate the reliability and quality of the 3D atomic structures through “all-atom contact analysis”, Ramachandran, complementary rotamer, covalent-geometry measures, optimization of hydrogen atoms, hydrogen bonds, steric clash, van der Waal interactions, Cβ deviation and MolProbity scores. All models provide the good quality cutoffs at percentiles above 66 (Table S1). All structural models were subjected for refinement by GalaxyWEB.^65^^,^^68^ The internal cavities detection was analyzed and illustrated by PyMOL with Cavity Detection Radius at 6 Solvent Radaii and Cavity Detection Cutoff at 5 Solvent Radii, respectively.

#### Molecular docking, visualization and validation

The ABCG2 homology models (both NBD and TMD) generated in this study were used for docking analysis. All ligands chemical structures (mitoxantrone, pheophorbide A, rhodamine 123, daunorubicin and Ko143) were obtained from PubChem database. Docking of ligands with ABCG2 variants was conducted using the CB-Dock (Cavity-detection guided Blind Docking).^39^^,^^40^ This unbiased computational approach considers protein surface curvatures, along with cavity detection through clustering and docking via AutoDock Vina. This methodology allows for the identification of five high-probability binding sites within ABCG2, including the dimensions of binding regions and the centers of the binding sites. The calculated docking scores reflecting binding energies at these sites, yield binding affinities for ligands at given residues. Lower binding energy values indicate a better fit for protein-ligand interactions. Following this approach, the settings for standard blind docking were adjusted according to the previous methodology outlined.^69^ The docking center is set by the spatial geometric center of heavy atoms. To define the dimensions of the docking box, the distances from the center to each atom along the x, y, and z axes were recorded. The largest distance in each direction was then doubled, with an additional 5 Å added to define the final size of the docking box. The interaction between ABCG2 and ligands were analysed and visualized using PyMOL and LigPlot+.^64^ To evaluate the reliability of docking strategy, we performed the docking of ABCG2 with the previously published ligands. Then the docking results were aligned with the available structures to compare the location of the binding site and interacting residues. The outputs confirmed the consistency of the docking results from this study with the original structural templates. *LogP* values (partition coefficient P) that indicate the equilibrium of a compound in the spontaneous partition between organic and aqueous phases were calculated using ALOGPS 2.1^66^ to estimate the ratio of lipophilicity to hydrophilicity. The structures of compound molecules were obtained from PubChem as sdf files and applied as an input for *LogP* calculation (Figure S1).

#### Plasmid construction and site-directed mutagenesis

All mutations were constructed in vectors pcDNA3.1(-)-FLAG-hABCG2 or pEGFPC1-hABCG2 plasmids that encodes the N-terminal FLAG or GFP-tagged ABCG2. Side-directed mutagenesis was applied to create mutants using Phusion High Fidelity DNA polymerase (Thermo Fisher Scientific) according to the manufacture’s manual. Parental DNA was removed by DpnI digestion before chemically transforming into *E. coli* (DH5α) for plasmid preparation. All plasmids were verified by DNA sequencing. The primers used in this study are indicated in the Table S3.

#### Mammalian cell culture and transfection

Human embryonic kidney cells (HEK293) were maintained in Dulbecco’s modified Eagle’s medium (DMEM) (Life Technologies, Rockville, MD, USA), supplemented with 5% (v/v) FBS with humidity at 37°C and 5% CO_2_. HEK293 cells were seeded onto a 6-well plate. After 1-day of culturing, transfection was performed using self-made poly-ethyleneimine (PEI, Polysciences Inc., Germany) with 2 μg of plasmids in 100 μl of Opti-MEM (Life Technologies, Rockville, MD, USA). Two days post-transfection, cells were used for subsequent experiments. For stable cell lines generation, HEK293 cells were seeded for 1 day prior to transfection. After 2 days transfection, cells were trypsinized and seeded into 10-cm dishes at density 50-500 cells per plate and continued cultured in DMEM medium supplemented with 0.9 g/l of G418. The medium was replaced twice per week until the single colonies were observed. The single colony was isolated by trypsin digestion using cloning cylinder into 24-well plate with medium containing G418. The screening of stable cell line clones employed three complementary methods, including assessing protein expression levels using Western blot analysis with an anti-ABCG2 antibody, quantifying efflux function by flow cytometry with specific substrates (mitoxantrone, pheophorbide A, and rhodamine 123) and the ABCG2-specific inhibitor (Ko143), and evaluating membrane localization of GFP-tagged ABCG2 variants by confocal microscopy.

#### Immunodetection

Immunoblotting was performed with standard protocols with minor modification suitable for membrane proteins. Cells were harvested after trypsin digestion and then centrifuged at 14,000 x g for 1 min at 4°C, followed by a washing with ice-cold PBS (Phosphate buffered saline, pH 7.4). Cell pellets were then lysed in ice-cold lysis buffer (50 mM Tris pH 8.0, 120 mM NaCl, 1 mM EDTA, 2% Triton X-100 and freshly added protease inhibitor cocktail (cOmplete Protease and Phosphate inhibitor Cocktails, Roch)). Cell debris was removed by centrifugation at 1,200 x g for 2 min at 4°C. The supernatants were collected and mixed with Laemmli sample buffer in the presence or absence of 100 mM DTT (dithiothreitol) before subjected to SDS-PAGE (8-10% (w/v) acrylamide). Following electroblotted onto nitrocellulose (GE Healthcare Life sciences, Freiburg, Germany), membranes were blocked in blocking solution, 5% BSA in TBST buffer (Tris-buffered saline containing 0.1% Tween-20), at room temperature for 1 h. The primary antibodies for human ABCG2 (mouse anti-ABCG2 antibody (BXP-21) (Santa Cruz Biotechnology, CA, USA)) or β-actin (rabbit anti-β-actin (D6A8) (Cell Signaling, MA, USA)) were diluted in TBST at dilutions of 1:500 and 1:1000, respectively. After the incubation at 4°C for overnight, membranes were washed with TBST for 15 min three times. Subsequently, the secondary antibodies against mouse or rabbit, IRDye® 800CW or 680CW (LI-COR Biosciences, Homburg, Germany) were prepared in TBST at dilution 1:10,000 and incubated at room temperature for 1 h. The signals were detected with the 800 or 680 channels of the Odyssey Imaging Systems and quantified by using Image Studio software version 2.1 (LI-COR® Biosciences, Homburg, Germany).

#### Confocal microscopy

The cover glass was coated with poly-L-lysine and put in 24-well plate prior to seeding. Subsequently, HEK293 cells were seeded and culture for overnight. Then cells were transfected with plasmid using PEI and Opti-MEM. After 2-day post transfection, culture medium was removed followed by washing with ice-cold PBS twice. Cells were then fixed with 4% formaldehyde in PBS at room temperature for 10 min before washing three times with PBS. Nuclei were stained using 5 μg/ml DAPI (4′,6-diamidino-2-pheny-lindole) in PBS at room temperature for 10 min. Following by washing with PBS for three times, cells were quenched by incubated in 100 mM glycine for 15 min at room temperature. The cover slip was then washed again before mounting on to microscopic slides with Fluoro Gel mounting medium prior to imaging by Zeiss LSM700 confocal microscopy. The images were analyzed using ZEN2012 program software.

#### Functional assay by flow cytometry

Accumulation of compound in ABCG2 variants were measured by flow cytometer FACSCalibur (Becton Dickinson, CA, USA). HEK293 cells were harvested by trypsin digestion followed by washing with ice-cold PBS and centrifugation at 1,000 x g at 4°C for 1 min. Supernatant was removed. Then the cell pellet containing 10^5^ cells was re-suspended in HPMI buffer (10 mM Hepes, 120 mM NaCl, 5 mM KCl, 400 μM MgCl_2_, 40 μM CaCl_2_, 10 mM NaHCO_3_, 10 mM glucose, 5 mM Na_2_HPO_4_, pH 7.4). Cells were pre-incubated with inhibitors or interest compounds for 5 min at 37°C followed by adding substrate (7 μM mitoxantrone, or 1 μM pheophorbide A or 0.5 μM rhodamine 123 or 1 μM daunorubicin) and further incubated for 20 min at 37°C. Consequently, cells were collected by washing with ice-cold PBS and centrifugation at 1,000 x g for 2 min at 4°C. Supernatant was removed. The zero-flux assay was performed in substrate-free buffer for further 20 min under the same condition. Then the reaction was stop by placing the reaction tube into ice-cold water for 5 min. Cells were centrifuged at 1,000 x g for 2 min at 4°C and followed by discard supernatant. Cell pellet was re-suspended in ice-cold PBS prior analysis by Flow cytometer. Intracellular accumulation was measured by FACSCalibur. Mitoxantrone, pheophorbide A and daunorubicin accumulation were evaluated with FL3 at excitation/emission wavelength at 488/670 nm while rhodamine 123 was measured with FL1 at excitation/emission wavelength at 488/534 nm. The population of viable cells were gated and collected for 10^4^ cells per data point. The signals of mitoxantrone, pheophorbide A and daunorubicin were measured in GFP-tagged ABCG2 variants with GFP-expressing cells to normalize the efflux function. The signal of rhodamine 123 is overlapped to FL1 by GFP, therefore, the accumulation of rhodamine 123 were analyzed in untagged-ABCG2. Data was analyzed using FlowJo Software Inc. (Stanford University).

#### Membrane protein preparation

HEK293 cells expressing ABCG2 variants or a Mock control were cultured and harvested. Cells were washed twice with ice-cold PBS, before collecting into a microtube by scraping. Cell pellets were recovered by centrifugation at 15,000 x g for 1 min at 4°C, followed by resuspension in ice-cold TMEP buffer (50 mM Tris pH7, 50 mM Mannitol, 2 mM EGTA, and protease inhibitor cocktail). Cell lysis was achieved by passing the suspension through a 27-gauge needle using a syringe for 20 times. The lysate was incubated on ice for 30 min for complete lysis. Subsequently, the lysate was centrifuged under 4°C at 500 x g for 10 min to remove debris, and the supernatants were collected in new tubes. The supernatants were centrifuged at 1,200 x g for 5 min to remove mitochondria, and resulting supernatants were transferred to fresh tubes prior to ultracentrifugation at 100,000 x g for 60 min at 4°C. Pellet containing membrane protein fraction was resuspended in cold TMEP buffer. The protein concentration was determined using the Bradford assay and protein concentrations were adjusted to 2 mg/ml in TMEP buffer. Aliquots of samples were stored at −80°C until further use.

#### ATPase activity assay

The vanadate-sensitive ATPase activity of ABCG2 was assessed using a membrane protein fraction from HEK293 cells. Five micrograms of membrane protein were incubated at 37°C for 10 min in ATPase assay buffer (50 mM MOPS, 50 mM KCl, 0.5 mM EGTA, 5 mM NaN_3_, and pH 7.0) in the presence or absence of 100 μM Na_3_VO_4_ (sodium orthovanadate). The reaction was initiated by adding 4 mM of ATP/Mg^2+^ to obtain a final volume of 50 μl and incubated at 37°C for 30 min. To terminate the reaction, 40 μl of 5% SDS was added, followed by 100 μl of color reagent (3.33% (v/v) H_2_SO_4_, 0.48% (w/v) ammonium molybdate, 0.006% (w/v) antimony potassium tartrate, 5.7% (v/v) acetic acid, and 0.24% (w/v) ascorbic acid). The mixture was incubated at room temperature for an additional 30 min, and released inorganic phosphate was detected using a microplate reader (VICTOR Nivo, Perkin-Elmer, Turku, Finland) at a wavelength of 750 nm. An SDS-treated sample served as the background control. Vanadate-sensitive ATPase hydrolysis was calculated by subtracting the phosphate release from the vanadate-treated sample from that of the control.

#### Figure preparation

All images of structural models were analyzed and conducted in PyMOL (The PyMOL Molecular Graphics System, version 2.5.2, Schrödinger). The schematic illustrations of drug compounds and ligands were prepared using LigPlot+.

### Quantification and statistical analysis

All values in this study are represented as mean with SEM, unless stated otherwise. All *in vitro* experiments were performed using at least 3 independent biological replicates. Figures and statistical analyses were generated and performed using Dunnett’s multiple comparison test by GraphPad Software Inc. (San Diego, CA, USA) Prism version 6.00.

### Additional resources

The authors confirm that all data underlying the findings are fully available without restrictions. All relevant data are within the paper and the supplemental information. The raw datasets, including coordinates of structural models in this study, are available at https://github.com/kakulab/ABCG2-CellReports.git.
