# Supplementary material for: The human ABCG2 transporter engages three gates to control multidrug extrusion
Source: iScience. 2025 Feb 28;28(3):112125. doi: 10.1016/j.isci.2025.112125 (PMC11957596; doi:10.1016/j.isci.2025.112125)
Supplement: Document S1. Figures S1–S13 and Tables S1–S3 [file mmc1.pdf]

iScience, Volume *28*

Supplemental information

The human ABCG2 transporter engages three gates to control multidrug extrusion

Narakorn Khunweeraphong and Karl Kuchler

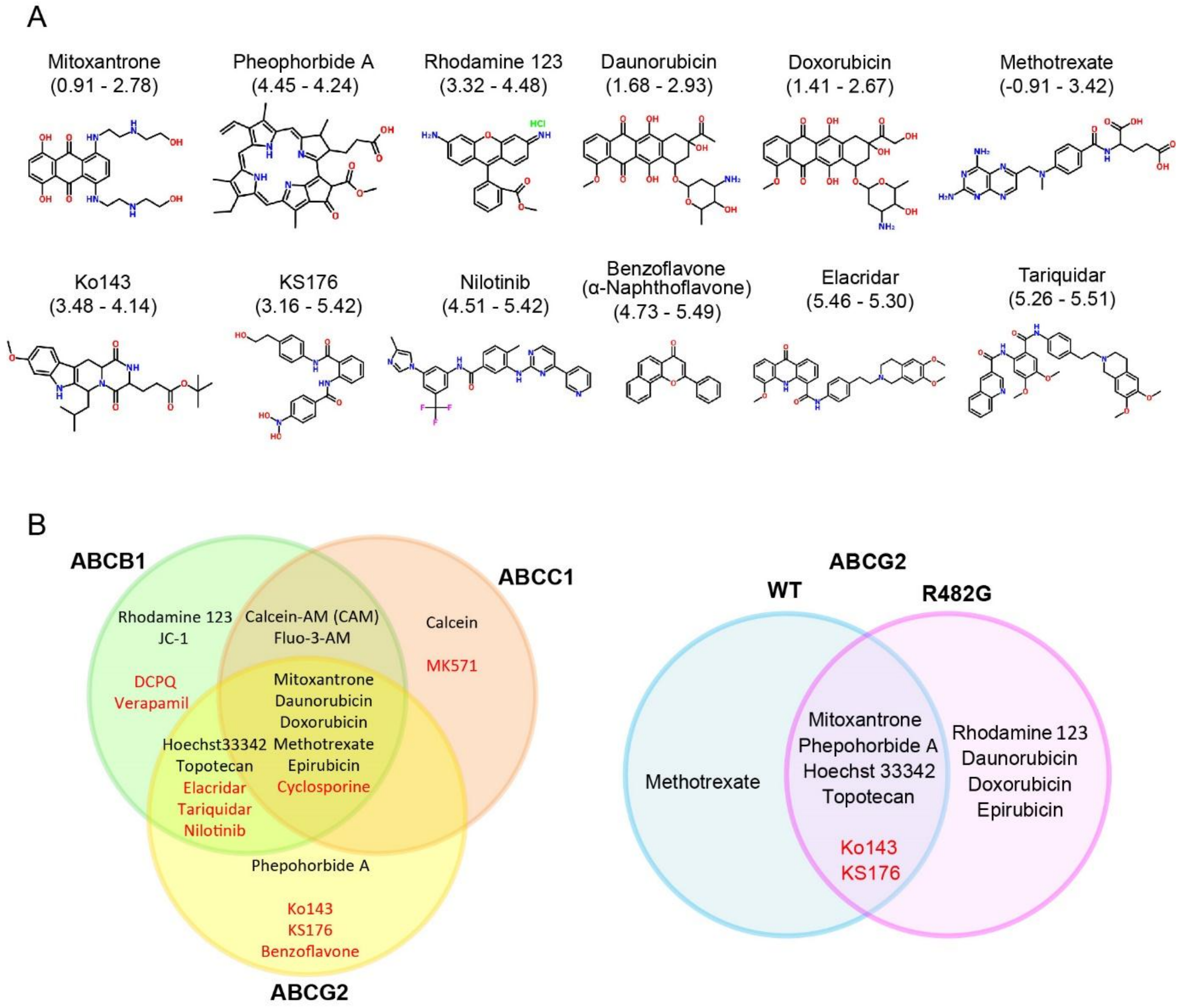

Figure S1. Substrates and inhibitors specificity in multidrug ABC transporters

(Related to INTRODUCTIONS and RESULTS)

(A) Chemical structures of ABCG2 substrates and inhibitors with LogP values in the corresponding brackets.

(B) The Venn diagrams represent the specificity and overlapping of substrates (black) and inhibitors (red) in three major multidrug resistance ABC transporters (left panel) and the change of drug specificity in ABCG2-R482G mutant (right panel). Substrates are indicated in black and inhibitors are in red, respectively.

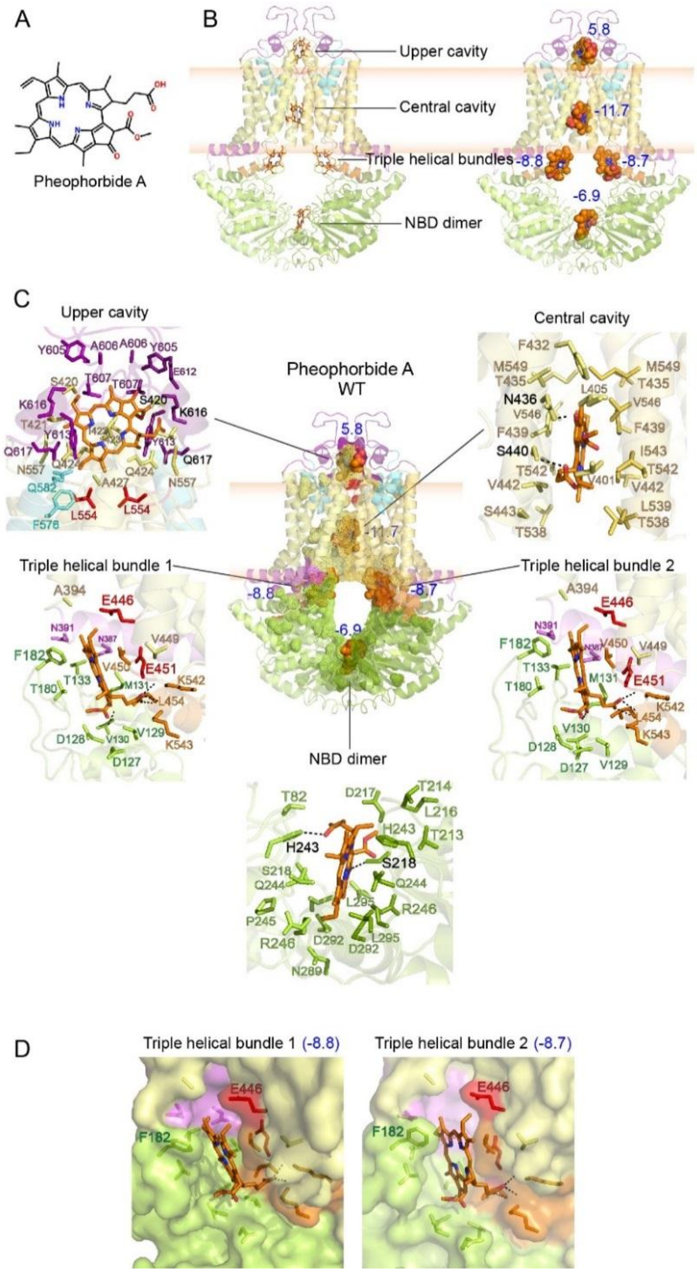

Figure S2. Molecular docking identifies five potential binding pockets for pheophorbide A and suggests the possible route for translocation pathway in ABCG2

(Related to Figure 2)

(A) Chemical structure of pheophorbide A.

(B) ABCG2 (transparent colored ribbon cartoon) in an inward-facing state (PDB ID: 6VXJ) provides five potential binding pockets for pheophorbide A (orange stick or space-filling, left or right panels, respectively) with their binding affinity score.

(C) Potential binding pockets in the internal cavities of ABCG2 with their corresponding interacting residues and binding affinity scores. The side-chains residues that form polar (H-bond) interactions are labelled in black while the non-polar (hydrophobic) interactions are indicated as color coded according to previous figure. Three essential negative residues at the triple helical bundle are colored in red.

(D) The ABCG2 substrate, pheophorbide A, is bound in the binding pocket close to the entry gate at triple helical bundle around transmission interface region. E446 (red) and F182 (green) provide side chains at the front of the entry gate

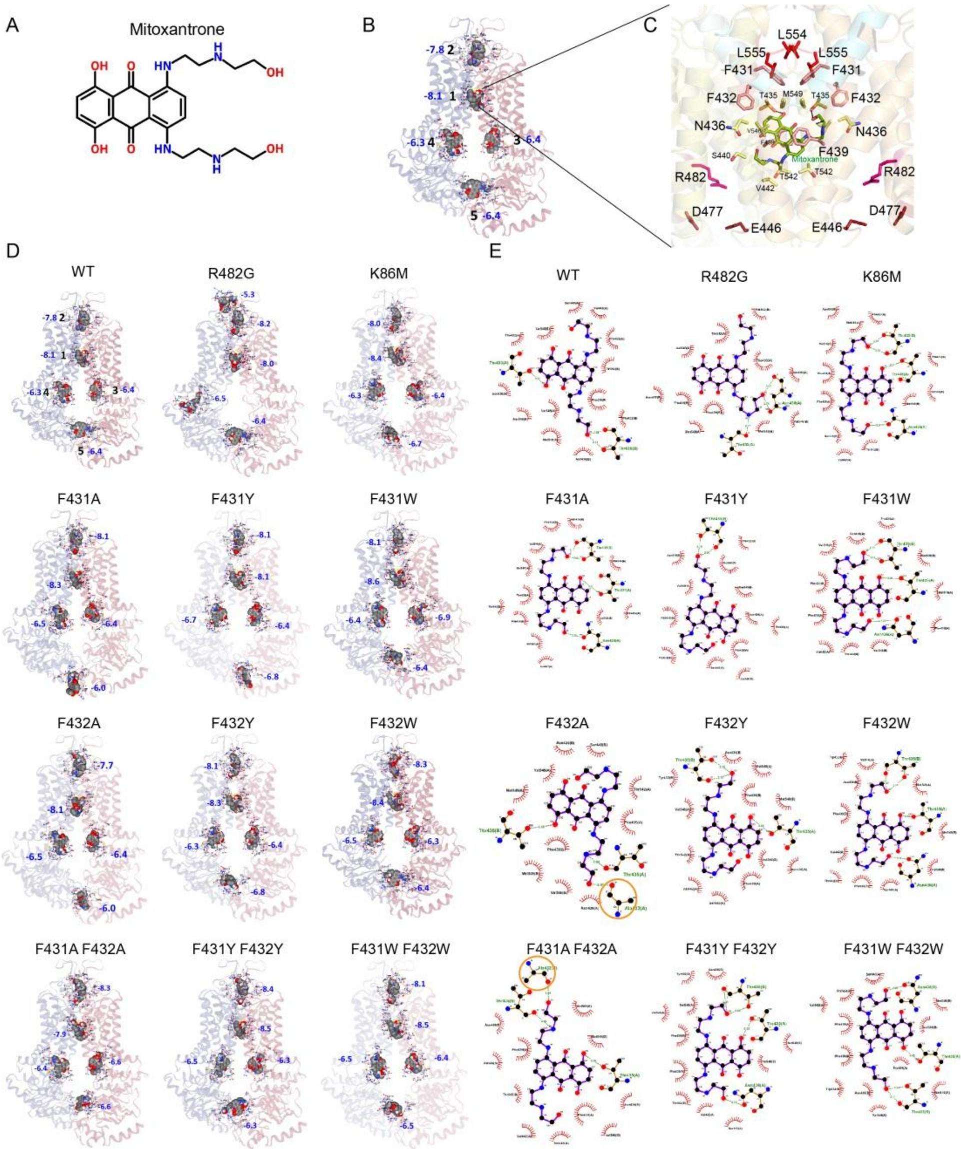

Figure S3. Docking of mitoxantrone in the central cavity in ABCG2 variants

(Related to Table 1)

(A) Chemical structure of mitoxantrone.

(B) Docking of ABCG2 (WT) homology model derived from 6VXJ-based with mitoxantorne was analyzed using CB-Dock and demonstrates top five binding sites with calculated binding affinity score. The two protomers are colored in blue and red, respectively. The molecules of ligands are displayed as space-filling.

(C) All relavant residues surounding mitoxantrone at the central cavity, including leucine valve (red), three conserved phenylalanines (F431, F432 and F439, salmon), R482 (magenta), intracellular gate (brick red).

(D) Docking of ABCG2 variants with mitoxantrone demonstrate top five binding pockets in the molecule with their binding affinity scores demonstrated by CB-Dock.

(E) Interacting residues of mitoxantrone in ABCG2 variants corresponding to panel (D) represented as 2D diagram by LigPlot+.

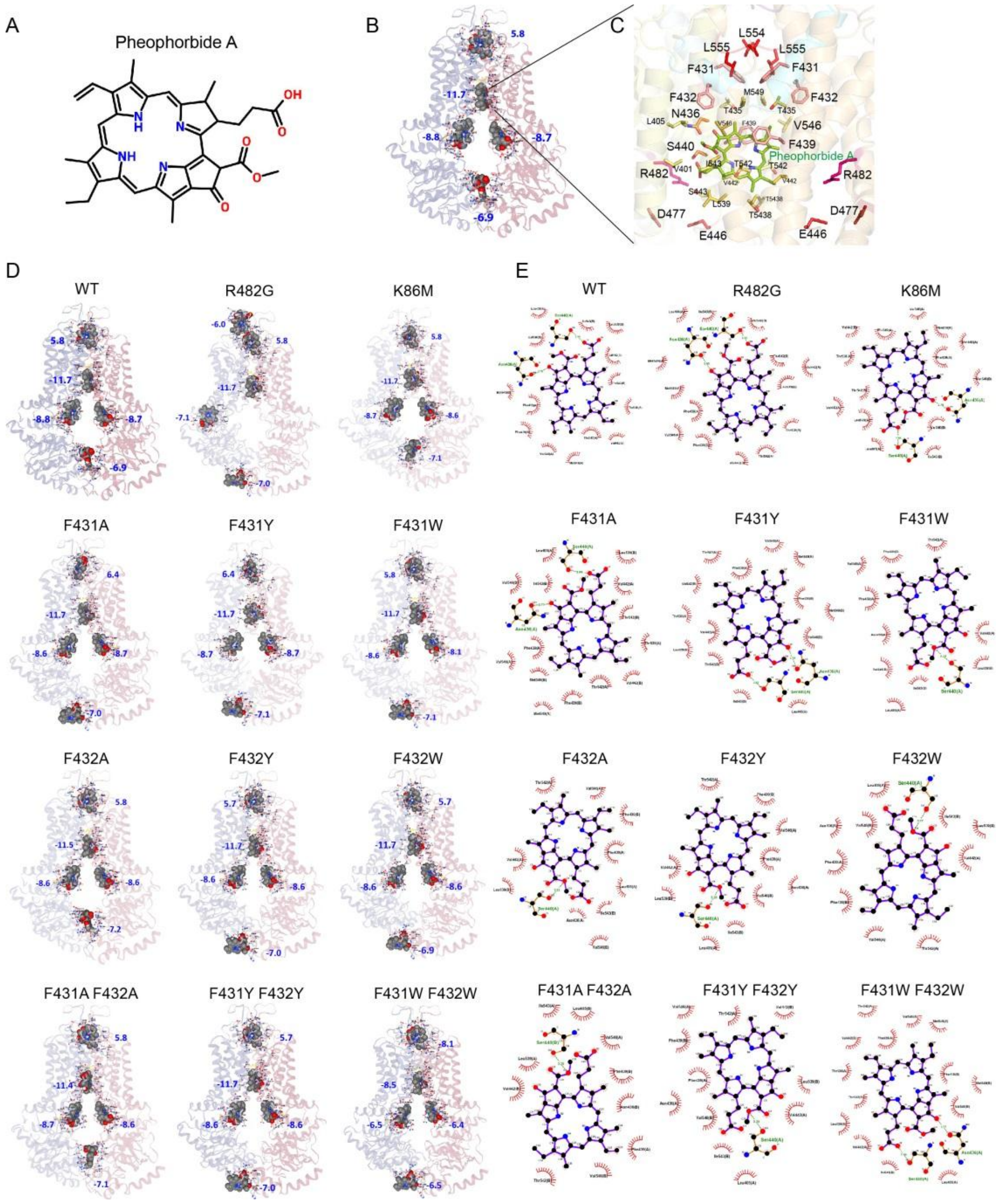

Figure S4. Docking of pheophorbide A in the central cavity in ABCG2 variants

(Related to Table 1 and Figure 3)

(A) Chemical structure of pheophorbide A.

(B) Docking of ABCG2 (WT) homology model derived from 6VXJ-based with pheophorbide A was analyzed and demonstrates top five binding sites with calculated binding affinity score. The two protomers are colored in blue and red, respectively. The molecules of ligands are displayed as space-filling.

(C) All relavant residues surounding pheophorbide A at the central cavity, including leucine valve (red), three conserved Phenylalanines (F431, F432 and F439, salmon), R482 (magenta), intracellular gate (brick red).

(D) Docking of ABCG2 variants with pheophorbide A demonstrate top five binding pockets in the molecule with their binding affinity scores.

(E) Interacting residues of pheophorbide A in ABCG2 variants corresponding to panel (D) represented as 2D diagram by LigPlot+.

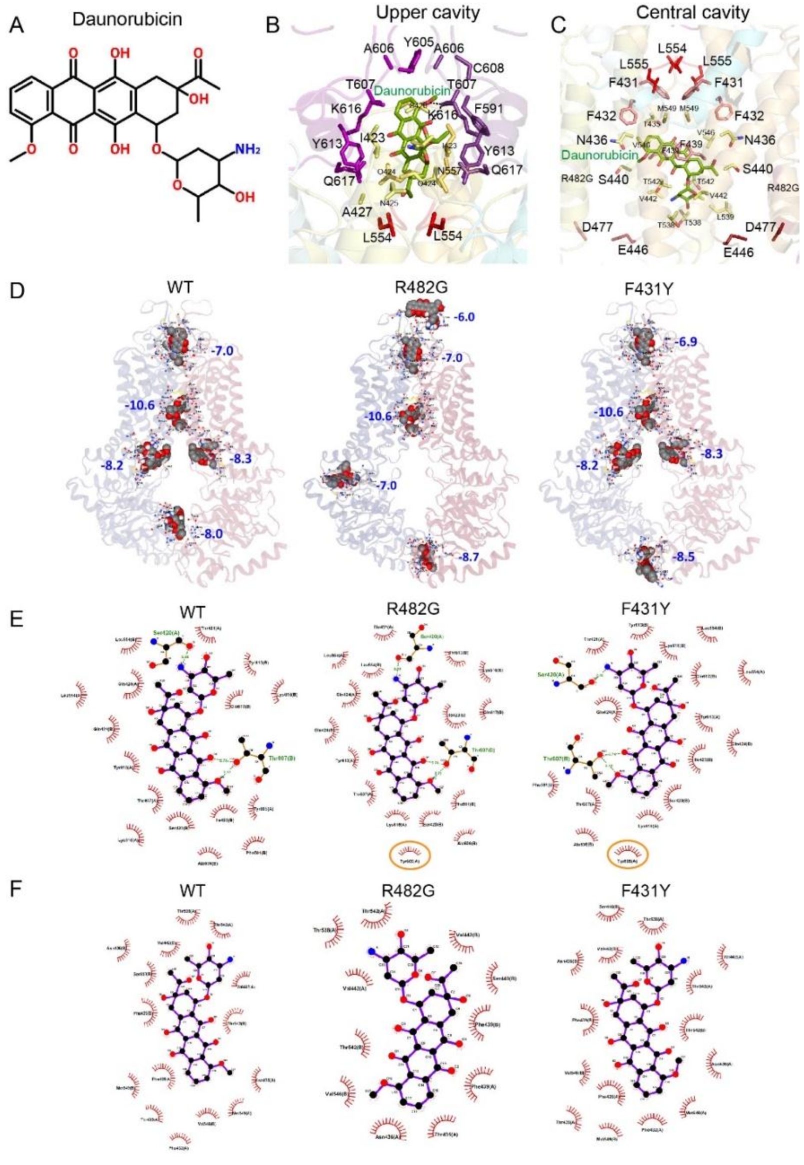

Figure S5. Docking of daunorubicin in the cavities of ABCG2 variants

(Related to Table 1 and Figure 3)

(A) Chemical structure of daunorubicin.

(B) Docking of daunorubidin binding in the upper cavity of ABCG2-R482G mutant represents all hydrophobic interaction residues as sticks. Colors are corresponding to their motifs. Leucine valve (red), TMH (yellow), extracellular loop 3 (purple). Rhodamine 123 is represented as a green stick.

(C) All relavant residues surounding Rhodamine 123 at the central cavity, including Leucine valve (red), three conserved Phenylalanines (F431, F432 and F439, salmon), R482G (magenta), intracellular gate (brick red).

(D) Docking of ABCG2 variants with daunorubicin demonstrate top five binding pockets in the molecule with their binding affinity scores.

(E) Binding of daunorubidin in the upper cavity of ABCG2 variants. Interacting residues of daunorubidin in ABCG2 variants are represented as 2D diagram by LigPlot+.

(F) Binding of daunorubidin in the central cavity of ABCG2 variants. Interacting residues of daunorubidin in ABCG2 variants are represented as 2D diagram by LigPlot+.

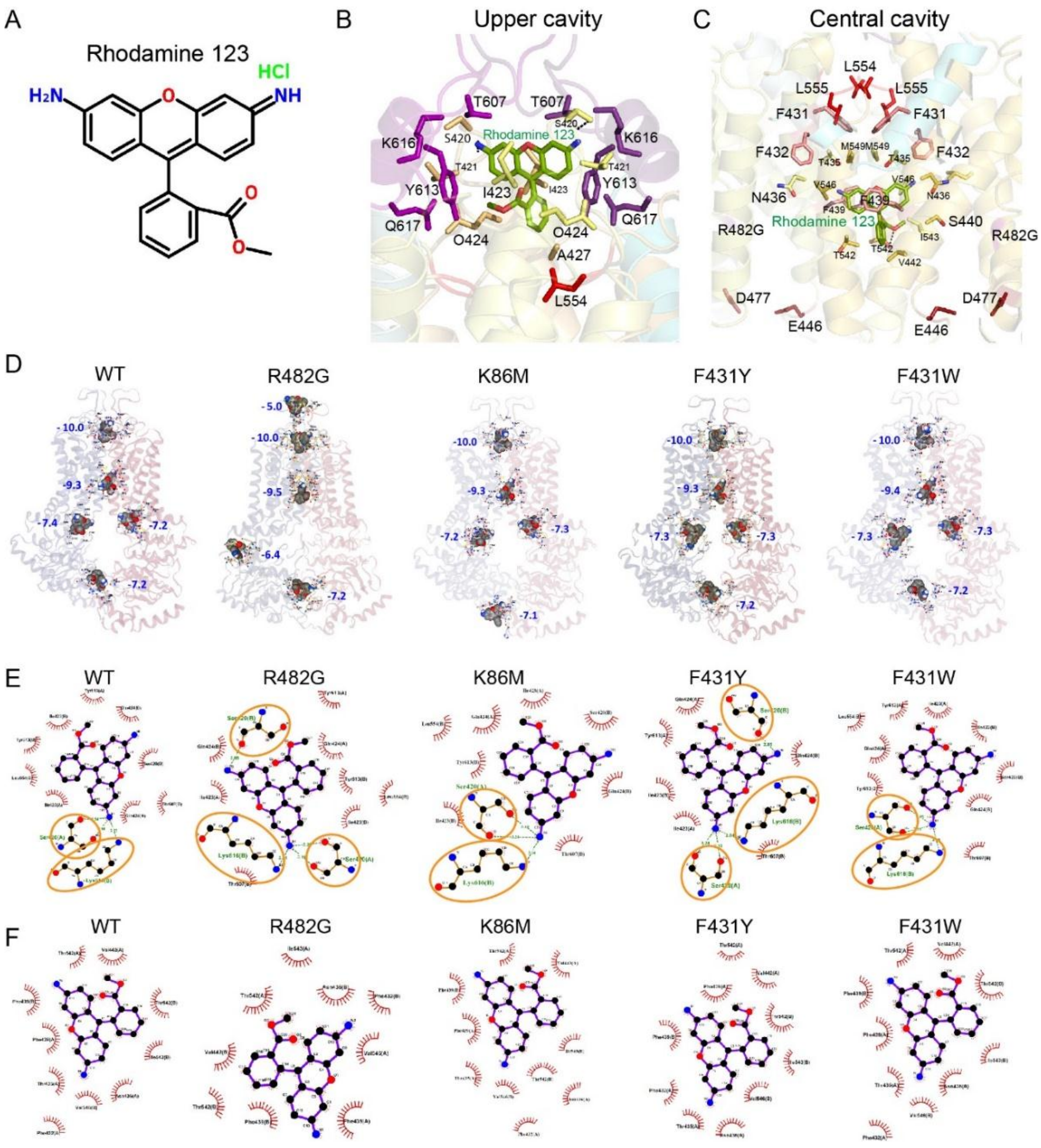

Figure S6. Docking of rhodamine 123 in the cavities of ABCG2 variants

(Related to Table 1 and Figure 3)

(A) Chemical structure of rhodamine 123.

(B) Docking of rhodamine 123 binding in the upper cavity of ABCG2-R482G mutant represents all hydrophobic interaction residues as sticks. Colors are corresponding to their motifs. Leucine valve (red), TMH (yellow), extracellular loop 3 (purple). Rhodamine 123 is represented as a green stick.

(C) All relavant residues surounding rhodamine 123 at the central cavity, including leucine valve (red), three conserved phenylalanines (F431, F432 and F439, salmon), R482G (magenta), intracellular gate (brick red).

(D) Docking of ABCG2 variants with rhodamine 123 demonstrate top five binding pockets in the molecule with their binding affinity scores.

(E) Binding of rhodamine 123 in the upper cavity of ABCG2 variants. Interacting residues of rhodamine 123 in ABCG2 variants are represented as 2D diagram by LigPlot+.

(F) Binding of rhodamine 123 in the central cavity of ABCG2 variants. Interacting residues of rhodamine 123 in ABCG2 variants are represented as 2D diagram by LigPlot+.

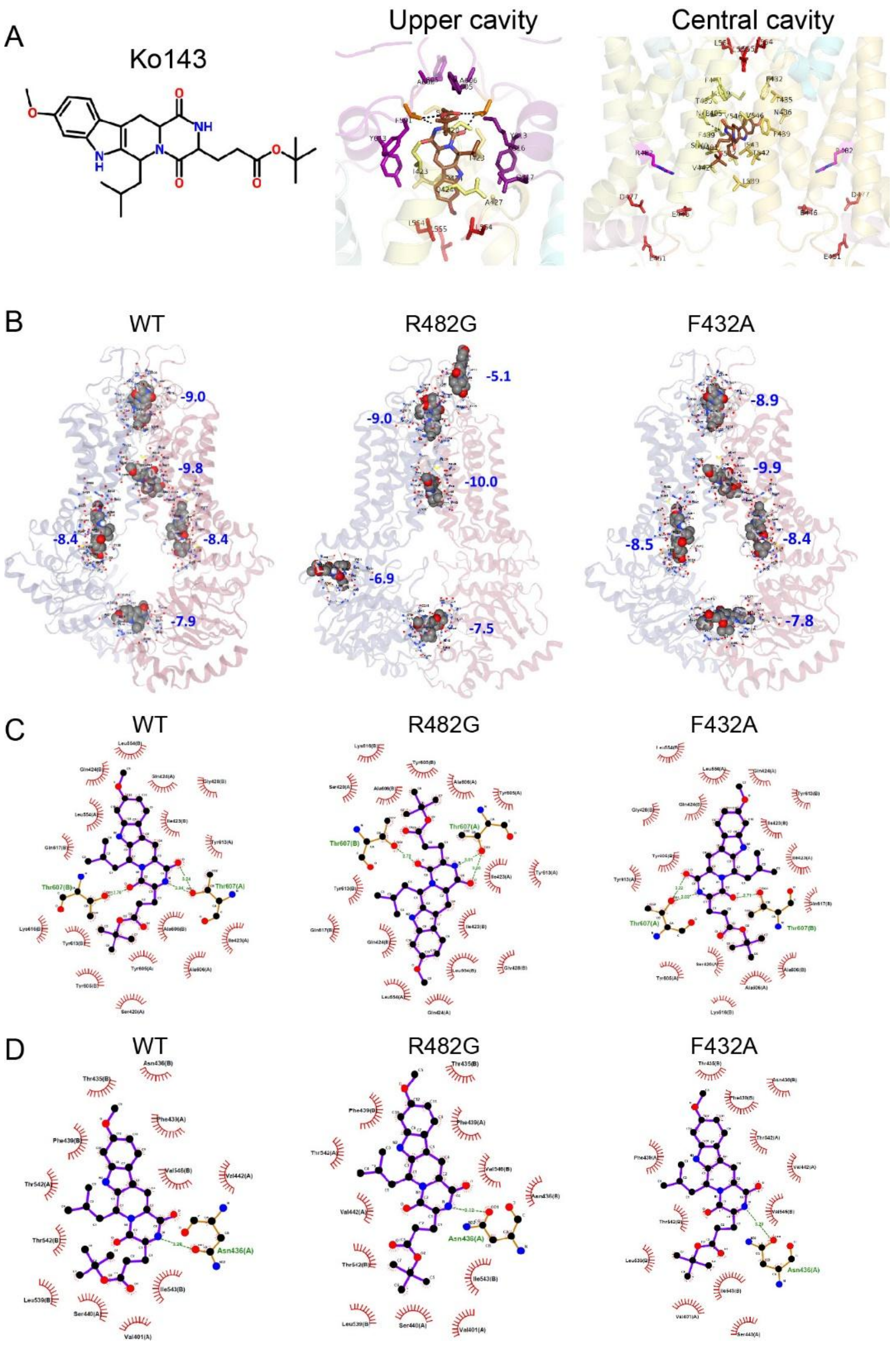

Figure S7. Docking of Ko143 in the cavities of ABCG2 variants

(Related to Table 1 and Figure 3)

(A) Chemical structure of Ko143.

(B) Docking of ABCG2 variants with Ko143 demonstrate top five binding pockets in the molecule with their binding affinity scores.

(C) Binding of Ko143 in the upper cavity of ABCG2 variants. Interacting residues of Daunorubidin in ABCG2 variants are represented as 2D diagram by LigPlot+.

(D) Binding of Ko143 in the central cavity of ABCG2 variants. Interacting residues of Daunorubidin in ABCG2 variants are represented as 2D diagram by LigPlot+.

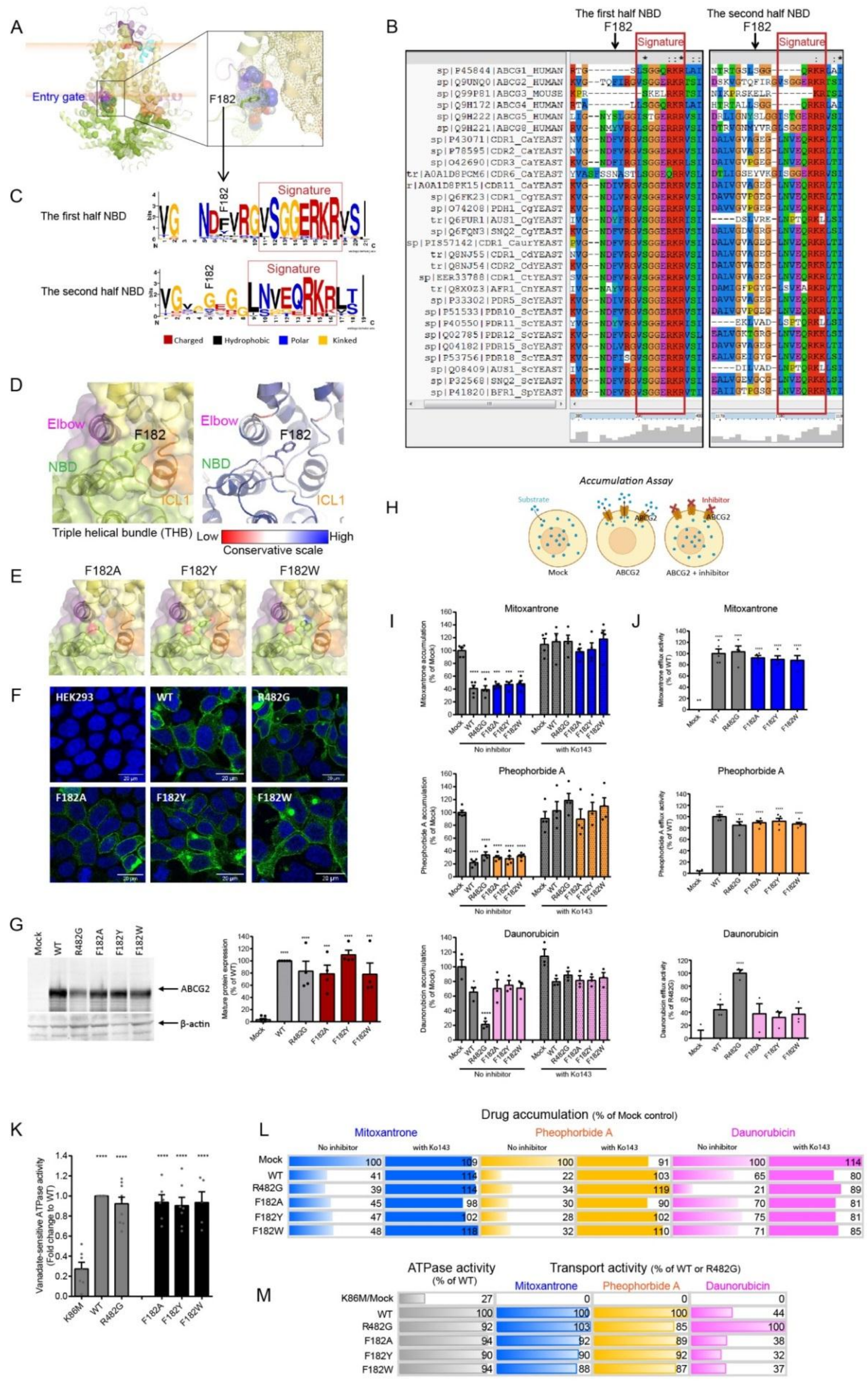
A
Entry gate
F182
B
The first half NBD
The second half NBD
F182
Signature
C
The first half NBD
The second half NBD
Signature
F182
Charged
Hydrophobic
Polar
Kinked
D
Elbow
F182
NBD
ICL1
Triple helical bundle (THB)
Low
High
Conservative scale
E
F182A
F182Y
F182W
F
HEK293
WT
R482G
F182A
F182Y
F182W
G
Mock
WT
R482G
F182A
F182Y
F182W
ABCG2
β-actin
Mature protein expression (% of WT)
H
Accumulation Assay
Substrate
Inhibitor
ABCG2
Mock
ABCG2
ABCG2 + inhibitor
I
J
Mitoxantrone
Mitoxantrone accumulation (% of Mock)
Mitoxantrone efflux activity (% of WT)
Pheophorbide A
Pheophorbide A accumulation (% of Mock)
Pheophorbide A efflux activity (% of WT)
Daunorubicin
Daunorubicin accumulation (% of Mock)
Daunorubicin efflux activity (% of R482G)
No inhibitor
with Ko143
K
Vanadate-sensitive ATPase activity (Fold change to WT)
K86M
WT
R482G
F182A
F182Y
F182W
L
Drug accumulation (% of Mock control)
Mitoxantrone
Pheophorbide A
Daunorubicin
No inhibitor
with Ko143
Mock 100 109 100 91 100 114
WT 41 114 22 103 65 80
R482G 39 114 34 119 21 89
F182A 45 98 30 90 70 81
F182Y 47 102 28 102 75 81
F182W 48 118 32 110 71 85
M
ATPase activity (% of WT)
Transport activity (% of WT or R482G)
Mitoxantrone
Pheophorbide A
Daunorubicin
K86M/Mock 27 0 0 0
WT 100 100 100 44
R482G 92 103 85 100
F182A 94 92 89 38
F182Y 90 90 92 32
F182W 94 88 87 37

Figure S8. F182 is not essential for ABCG2 function

(Related to Figure 2)

(A) F182 is located at the front of the binding pocket close to the entry gate around transmission interface.

(B) Amino acid sequence alignment of mammalian ABCGs with the first and second half of yeast PDRs. The signature motifs are highlighted in the red boxes. F182 residues of ABCG2 monomer are indicated corresponding to both protomers.

(C) Logo plots represent the relative frequency of each residue in around residue F182. The properties of the residues are indicated as colors code, charged (red), hydrophobic (black), polar (blue) and kinked (yellow), respectively.

(D) Zoom-in side view shows side chain of F182 at triple helical bundle (left panel) with the conservation analysis (right panel). Motifs are coloring as previous description, NBD (green), elbow helix (pink) and ICL1 (orange), respectively. The conservation scale is given as color-gradient: low conservation (red) to high conservation (blue).

(E) Mutational variants at 182 from phenylalanine to alanine, tyrosine or tryptophan change the structure at the front of entry gate.

(F) Membrane localization of GFP-tagged ABCG2 variants visualized to detect GFP-ABCG2 variants (green) by confocal microscopy. Nuclear DNA was stained with DAPI (blue). Microscopy data are from duplicated experiments. Scale bar in microscopy images correspond to 20 µm.

(G) Protein expression of ABCG2 variant by western blot (left panel) using the monoclonal anit-ABCG2 (BXP-21) antibody. β-actin was used as an internal loading control were quantified by Odyssey-based system independent experiments (n = 4-5). The signal of protein expression were individually normalized to β-actin and represented as a relative percentage of WT control (right panel).

(H) Cartoon represents the accumulation assay to investigate efflux function of ABCG2.

(I) Accumulation assay of ABCG2 variants with mitoxantrone (top, blue), pheophorbide A (middle, orange) and daunorubicin (bottom, pink) in the presence and absence of ABCG2-specific inhibitor, Ko-143, (n = 3-5). Accumulation level was normalized from GFP-expressing cells and represented as percentage to the mock control.

(J) Efflux function of ABCG2 variants with mitoxantrone (top, blue), pheoporbide A (middle, orange) and daunorubicin (bottom, pink), (n = 3-5). The efflux activity were normalized from GFP-expressing cells and represented as percentage to WT (mitoxantrone and pheophorbide A efflux) or R482G (daunorubicin efflux) as the control.

(K) Vanadate-sensitive ATPase activities of ABCG2 variants expressed in HEK293 cells. Grey dots represent values from independent biological replicates (n = 5-12) using 4 different batches of membrane preparations. Data are given as fold changes relative to WT.

(L) Intracellular accumulation of drugs levels, mitoxantrone (blue), pheophorbide A (yellow) and daunorubicin (pink), in HEK293 cells expressing ABCG2 variants in the absence (gradient fill) and presence (solid fill) of ABCG2-specific inhibitor (Ko143). Color bars represented as percentage of Mock control.

(M) Bar graph shows the relative ATPase activities (gray) and transport function of drugs related to panel (L). Color bars are shown as percentage activities related to WT (for ATPase activity, efflux of mitoxantrone and pheophorbide A) or R482G (efflux of daunorubicin), respectively.

All data in Figures G, I, J and K are shown as means ± SEM; ****$P < 0.0001$ and ***$P < 0.001$, **$P < 0.01$, *$P < 0.1$ vs. negative control of empty plasmid transfected HEK293 (mock) or K86M (ATPase-dead mutant) using Dunnett's multiple comparisons test for statistical analysis. Black dots refer to values from each independent experiment. Gray bars represent control groups. Colored bars demonstrated the variants.

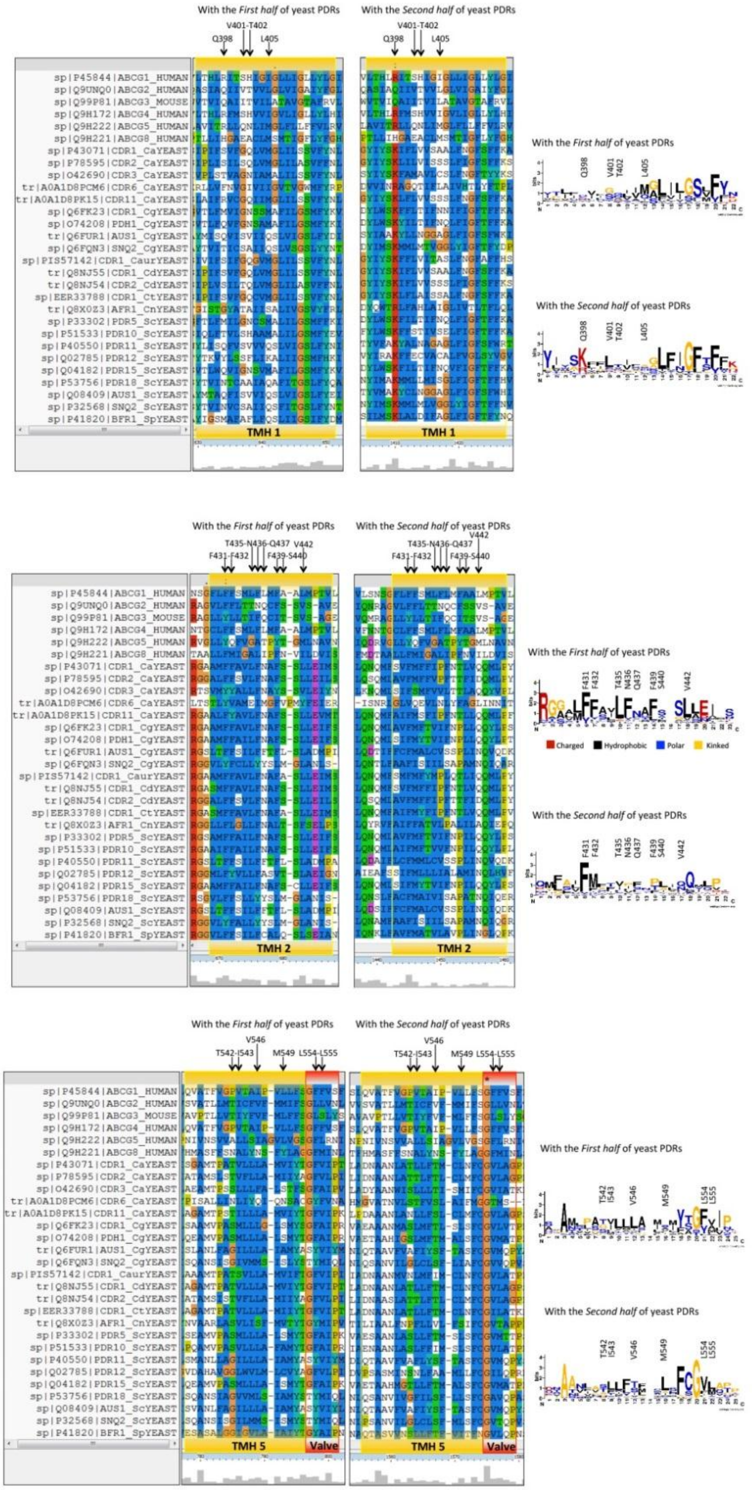

Figure S9. Conservation of residues at the central binding pocket of human ABCGs and yeast PDRs
(Related to Figure 4 and Figure 6)
The multiple sequence alignments (MSA) of amino acid residues in TMH1-TMH2-TMH5 of human ABCG2 were subjected to analyze with both protomers of yeast PDRs by using BioEdit and ClustalX2. The conserved residues are highlighted with the conservation scale as the height of gray bars at the bottom of each residue.

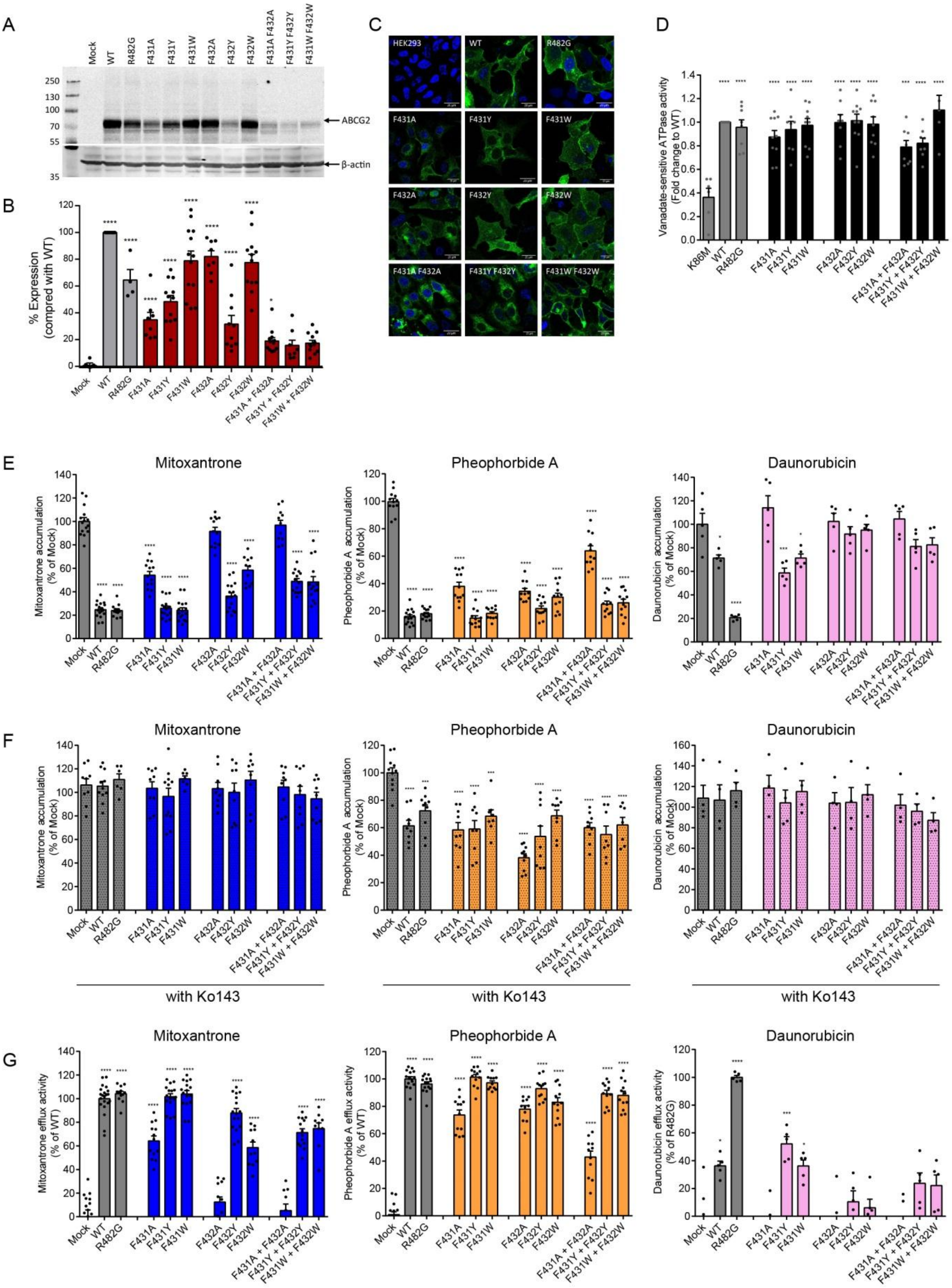
A
Mock WT R482G F431A F431Y F431W F432A F432Y F432W F431A F432A F431Y F432Y F431W F432W
250 130 100 70 55 35
ABCG2
β-actin
B
% Expression (compred with WT)
Mock WT R482G F431A F431Y F431W F432A F432Y F432W F431A + F432A F431Y + F432Y F431W + F432W
C
HEK293 WT R482G F431A F431Y F431W F432A F432Y F432W F431A F432A F431Y F432Y F431W F432W
D
Vanadate-sensitive ATPase activity (Fold change to WT)
K86M WT R482G F431A F431Y F431W F432A F432Y F432W F431A + F432A F431Y + F432Y F431W + F432W
E
Mitoxantrone
Mitoxantrone accumulation (% of Mock)
Pheophorbide A
Pheophorbide A accumulation (% of Mock)
Daunorubicin
Daunorubicin accumulation (% of Mock)
F
Mitoxantrone
Pheophorbide A
Daunorubicin
with Ko143
with Ko143
with Ko143
G
Mitoxantrone
Mitoxantrone efflux activity (% of WT)
Pheophorbide A
Pheophorbide A efflux activity (% of WT)
Daunorubicin
Daunorubicin efflux activity (% of R482G)

Figure S10. Expression and localization of F431-F432 variants

(Related to Figure 5)

(A) Western blotting of cell lysates from ABCG2 variants after transient transfected into HEK293 cells using the monoclonal antibodies specific for human ABCG2 (BXP-21) and β-actin (D6A8). Mature FLAG-tagged ABCG2 proteins migrates at approximately 75 kDa, while immature unglycosylated ABCG2 migrates just below. β-actin was applied as an internal loading control and migrate at approximately 45 kDa.

(B) Relative quantification of mature ABCG2 protein by immunoblotting. The signals from mature ABCG2 variants and β-actin on immunoblots were quantified by densitometry. ABCG2 levels were individually normalized to β-actin and data are represented as a percentage relative to WT from independent repeats (n = 4-13).

(C) Membrane localization of GFP-tagged ABCG2 variants under confocal microscopy (Ziess 710) to detect GFP-ABCG2 mutants (green). Nuclei was stained with DAPI (blue). Microscopic data are from tripicate experiments. Scale bar in the images corresponds to 20 μm.

(D) Vanadate-sensitive ATPase activities of ABCG2 variants expressed in HEK293 cells. Data are from 4 different batches of membrane preparations and given as fold changes relative to WT.

(E) Drug accumulation assays of ABCG2 variants with mitoxantrone (blue), pheophorbide A (orange), and daunorubicin (pink) in the absence of the ABCG2-specific inhibitor, Ko143 and quantified by flow cytometry in a FACS Calibur (n = 4-20). Intracellular drug accumulation levels were normalized from GFP-expressing cells and represented as percentage to the mock control.

(F) Drug accumulation assays of ABCG2 variants in the presence of Ko143.

(G) Efflux activities of ABCG2 variants with distinct drug substrates; mitoxantrone (blue), pheophorbide A (orange) and daunorubicin (pink) were performed in HEK293 cell transfected with GFP-ABCG2 variants. Data were normalized from GFP-expressing cells and represented as percentage to WT (mitoxantrone and pheophorbide A efflux) or R482G (daunorubicin efflux) as controls (n = 4-20).

Data in Figures B, D, E, F and G are shown as means $\pm$ SEM; *****P* < 0.0001 and ****P* < 0.001, ***P* < 0.01, **P* < 0.1 vs. negative control of mock or K86M (ATPase-dead mutant) using Dunnett's multiple comparisons test for statistical analysis. Black dots refer to values from each independent experiment. Gray bars represent control groups. Colored bars demonstrated the variants.

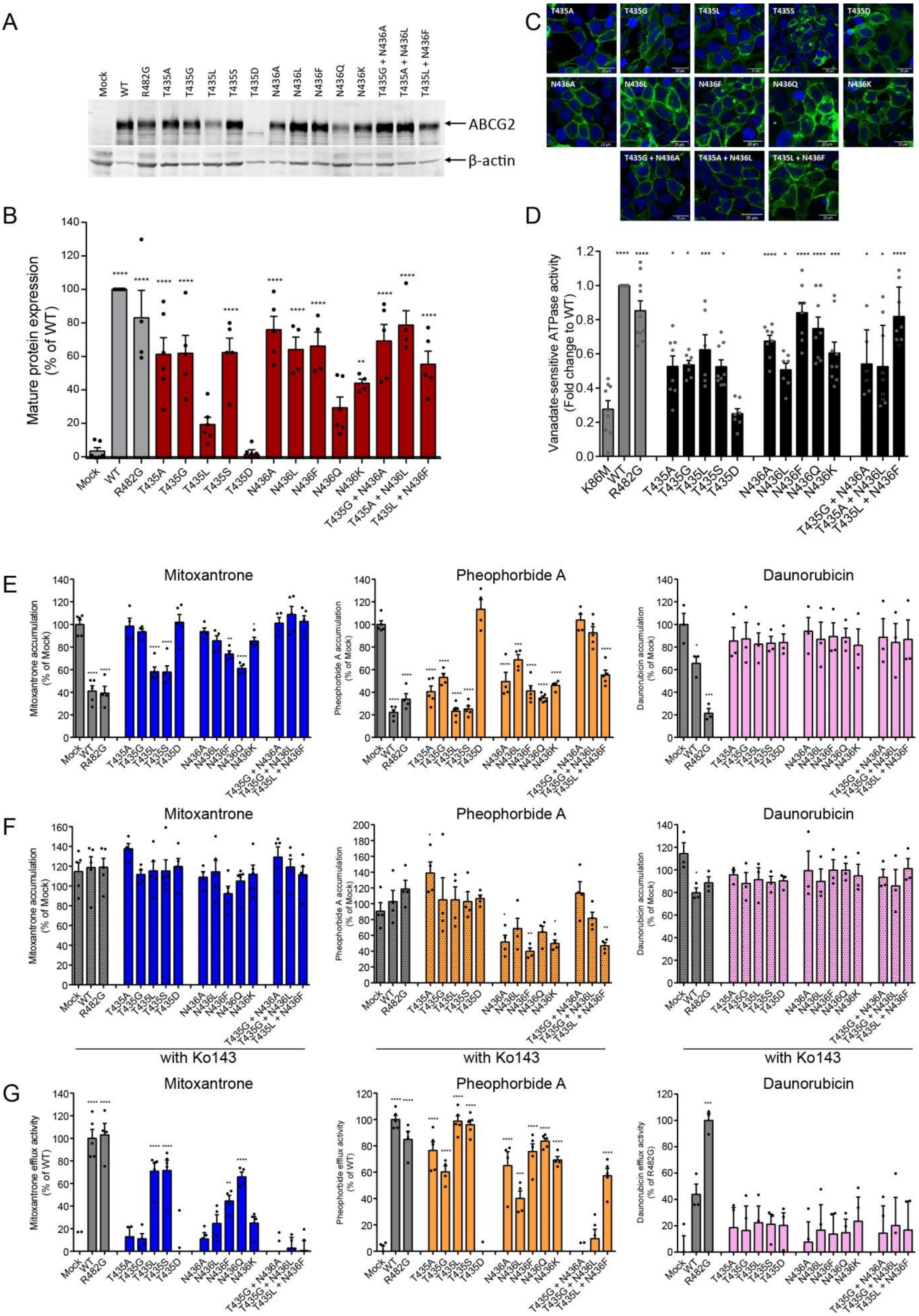
A
Mock
WT
R482G
T435A
T435G
T435L
T435S
T435D
N436A
N436L
N436F
N436Q
N436K
T435G + N436A
T435A + N436L
T435L + N436F
ABCG2
β-actin
B
Mature protein expression (% of WT)
C
T435A
T435G
T435L
T435S
T435D
N436A
N436L
N436F
N436Q
N436K
T435G + N436A
T435A + N436L
T435L + N436F
D
Vanadate-sensitive ATPase activity (Fold change to WT)
K86M
E
Mitoxantrone
Pheophorbide A
Daunorubicin
Mitoxantrone accumulation (% of Mock)
Pheophorbide A accumulation (% of Mock)
Daunorubicin accumulation (% of Mock)
F
with Ko143
G
Mitoxantrone efflux activity (% of WT)
Pheophorbide efflux activity (% of WT)
Daunorubicin efflux activity (% of R482G)

Figure S11. Expression and localization of T435-N436 variants

(Related to Figure 6)

(A) Western blotting of cell lysates from ABCG2 variants after transient transfected into HEK293 cells using the monoclonal antibodies specific for human ABCG2 (BXP-21) and β-actin (D6A8). Mature FLAG-tagged ABCG2 proteins migrates at approximately 75 kDa, while immature unglycosylated ABCG2 migrates just below. β-actin was applied as an internal loading control and migrate at approximately 45 kDa.

(B) Relative quantification of mature ABCG2 protein by immunoblotting. The signals from mature ABCG2 variants and β-actin on immunoblots were quantified by densitometry. ABCG2 levels were individually normalized to β-actin and data are represented as a percentage relative to WT from independent repeats (n = 4-5).

(C) Membrane localization of GFP-tagged ABCG2 variants under confocal microscopy (Ziess 710) to detect GFP-ABCG2 mutants (green). Nuclei was stained with DAPI (blue). Microscopic data are from tripicate experiments. Scale bar in the images corresponds to 20 µm.

(D) Vanadate-sensitive ATPase activities of ABCG2 variants. Data are from independent experiments using 4 different batches of membrane preparations (n = 5-12) and shown as fold changes relative to WT.

(E) The accumulation assays of ABCG2 variants with mitoxantrone (blue), pheophorbide A (orange), and daunorubicin (pink) in the absence of ABCG2-specific inhibitor, Ko143, (n = 4-20). Accumulation level was normalized from GFP-expressing cells and represented as percentage to the mock control.

(F) The accumulation assays of ABCG2 variants in the presence of Ko143, (n = 4-20).

(G) The efflux activity of ABCG2 variants with mitoxantrone (blue), pheophorbide A (orange) and daunorubicin (pink) were done in HEK293 cell transfected with GFP-ABCG2 variants and measured by flow cytometer (FACSCalibur). Results were normalized from GFP-expressing cells and represented as percentage to WT (mitoxantrone and pheophorbide A efflux) or R482G (daunorubicin efflux) as the control (n = 4-20).

All data in Figures B, D, E, F and G are shown as means ± SEM; *****P* < 0.0001 and ****P* < 0.001, ***P* < 0.01, **P* < 0.1 vs. negative control of mock or K86M (ATPase-dead mutant) using Dunnett's multiple comparisons test for statistical analysis. Black dots refer to values from each independent experiment. Gray bars represent control groups. Colored bars demonstrated the variants.

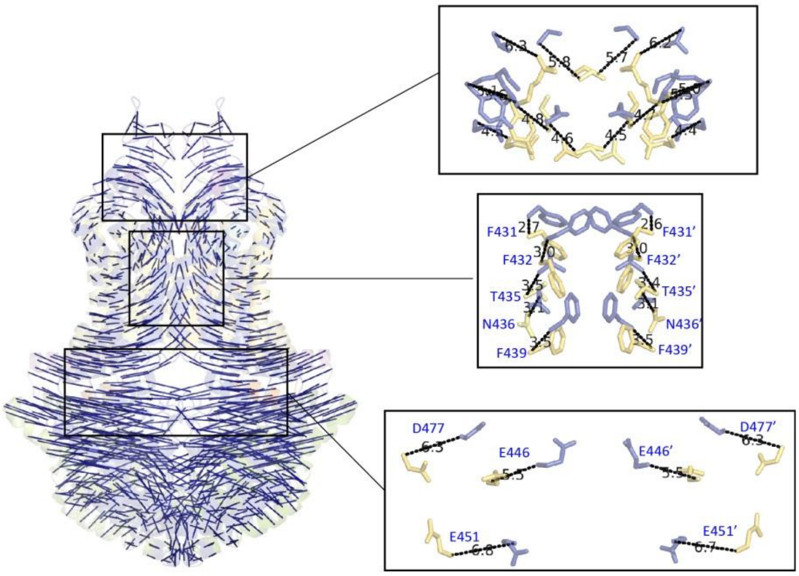

Figure S12. Movement of residues during conformational changes

(Related to Figure 4)

Left: structural alignment of ABCG2 in an inward-facing (IF) state (color-code yellow ribbon) compared to an outward-facing (OF) state (blue ribbon). Directions of movements are indicated by blue lines. The right panel with black boxes indicates the movement direction for relevant residues at the transmission interface (bottom box), central cavity (middle box), and upper cavity (upper box). Dotted lines illustrate the length of backbone movement in Angstroms (Å).

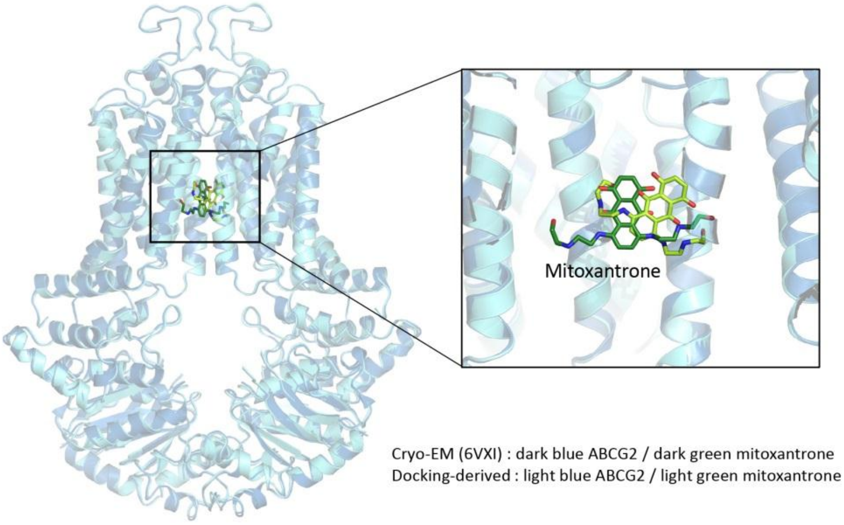

Figure S13. Validation of Docking result with cryo-EM structure

(Related to Figure 2)

Superimposition of the cryo-EM ABCG2 structures, 6VXI (mitoxantrone-bound: dark blue ribbon with dark green mitoxantrone) and the docking-derived structure (light blue ribbon struture with light green mitoxantrone), revealing an almost superimposable binding sites within the central cavity.

MolProbity		All-Atom Contacts	Protein Geometry									Peptide Omegas		Low-resolution Criteria		Additional validations	
original PDB	Variants	Clashscore, all atoms (Percentile)	Poor rotamers (%)	Favoured rotamers (%)	Ramachandran outliers (%)	Ramachandran favored (%)	Rama distribution Z-score	MolProbity score (Percentile)	Cβ deviations > 0.25 Å	Bad bonds (%)	Bad angless (%)	Cis Prolines	Twished Peptides (%)	CaBLAM outlines (%)	CA Geometry outliers (%)	Chiral volume outliers (%)	Water with clashes (%)
6VXJ	WT	100th	3.42	86.20	0.00	90.50	-5.74	99th	0.00	0.00	0.00	0.00	0.18	5.10	0.73	0/1410	0.00
6VXI		100th	2.07	85.89	0.18	90.32	-6.00	100th	0.00	0.00	0.02	0.00		5.60	0.18	0/1410	0.00
6HZM		100th	0.40	91.67	0.00	95.57	-5.46	100th	0.00	0.11	0.15	0.00	0.69	3.10	0.44	0/1466	50.00
6HBU		100th	0.00	97.79	0.52	94.62	-5.40	100th	0.00	0.09	0.17	0.00	0.86	3.50	0.70	0/1466	0.00
6VXF		100th	1.25	88.25	0.18	92.10	-4.65	100th	0.00	0.00	0.03	0.00		6.00	0.00	0/1408	0.00
6VXH		100th	0.42	85.76	0.18	89.95	-6.42	100th	0.00	0.03	0.09	0.00	0.09	6.70	1.28	0/1424	0.00
6HCO		97th	0.30	96.28	0.00	92.07	-4.59	100th	0.00	0.11	0.08	0.00	0.13	4.00	0.39	0/1954	0.00
6ETI		100th	0.15	96.74	0.19	94.48	-4.70	100th	0.00	0.11	0.06	0.00	0.13	3.90	0.39	0/1968	0.00
6FEQ		100th	0.00	96.96	0.00	94.22	-4.17	100th	0.00	0.13	0.07	0.00	0.13	3.30	0.46	0/1952	0.00
6HIJ		100th	0.00	98.77	0.18	96.28	-3.33	100th	0.00	0.14	0.17	0.00	0.18	2.30	0.00	0/1520	0.00
6FFC		100th	0.00	98.77	0.18	95.92	-3.27	100th	0.00	0.15	0.03	0.00	0.18	2.20	0.00	0/1432	0.00
5NJ3		97th	0.68	95.79	0.10	93.89	-3.80	100th	0.00	0.03	0.07	13.64	0.41	2.60	0.84	0/2152	0.00
7NFD		89th	0.44	99.04	0.13	83.95	-5.50	98th	0.07	0.02	0.06	0.00	0.25	5.90	0.91	0/1960	0.00
7NEQ		96th	5.78	71.33	0.00	92.11	-3.70	91th	0.00	0.02	0.01	0.00		4.40	0.39	0/1977	6.25
7NEZ		97th	0.82	95.39	0.00	92.98	-2.47	100th	0.21	0.20	0.05	0.00		4.00	0.39	0/1951	0.00
7OJ8		100th	0.61	95.21	0.53	94.18	-1.11	100th	0.00	0.33	0.30	0.00		2.30	0.36	0/1478	0.00
7OJH		100th	0.52	96.49	0.00	97.15	0.37	100th	0.00	0.46	0.23	0.00		2.00	0.36	0/1441	0.00
7OJI		100th	0.41	97.13	0.18	95.04	-0.15	100th	0.00	0.45	0.23	0.00	0.18	1.80	0.18		0.00
8BHT		100th	0.41	94.01	0.18	97.50	0.30	100th	0.00	0.11	0.13	0.00		1.10	0.00	0/1438	0.00
8BI0		100th	0.20	95.59	0.18	96.35	0.83	100th	0.00	0.11	0.11	0.00		2.00	0.18	0/1466	0.00

MolProbity		All-Atom Contacts	Protein Geometry									Peptide Omegas		Low-resolution Criteria		Additional validations	
Structures	Variants	Clashscore, all atoms (Percentile)	Poor rotamers (%)	Favoured rotamers (%)	Ramachandran outliers (%)	Ramachandran favored (%)	Rama distribution Z-score	MolProbity score (Percentile)	Cβ deviations > 0.25 Å	Bad bonds (%)	Bad angless (%)	Cis Prolines	Twished Peptides (%)	CaBLAM outlines (%)	CA Geometry outliers (%)	Chiral volume outliers (%)	Water with clashes (%)
6VXJ based homology models	WT	89th	3.42	86.20	0.00	90.50	-5.74	58th	0.00	0.00	0.00	0.00	0.18	5.10	0.73	0/1410	0.00
	R482G	88th	3.43	86.17	0.00	90.50	-5.73	58th	0.00	0.00	0.00	0.00	0.18	5.10	0.73	0/1408	0.00
	F431A	88th	3.43	86.17	0.00	90.50	-5.74	58th	0.00	0.00	0.00	0.00	0.18	5.10	0.73	0/1410	0.00
	F431Y	86th	3.42	86.20	0.00	90.50	-5.74	57th	0.00	0.00	0.00	0.00	0.18	5.10	0.73	0/1410	0.00
	F431W	82th	3.42	86.20	0.00	90.50	-5.74	54th	0.00	0.00	0.00	0.00	0.18	5.10	0.73	0/1410	0.00
	F432A	88th	3.43	86.17	0.00	90.50	-5.74	58th	0.00	0.00	0.00	0.00	0.18	5.10	0.73	0/1410	0.00
	F432Y	86th	3.42	86.20	0.00	90.50	-5.74	57th	0.00	0.00	0.00	0.00	0.18	5.10	0.73	0/1410	0.00
	F432W	77th	3.42	86.20	0.00	90.50	-5.74	52th	0.00	0.00	0.00	0.00	0.18	5.10	0.73	0/1410	0.00
	AA	88th	3.44	86.15	0.00	90.50	-5.75	58th	0.00	0.00	0.00	0.00	0.18	5.10	0.73	0/1410	0.00
	YY	84th	3.42	86.20	0.00	90.50	-5.75	56th	0.00	0.00	0.00	0.00	0.18	5.10	0.73	0/1410	0.00
	WW	70th	3.42	86.20	0.00	90.50	-5.74	49th	0.00	0.00	0.00	0.00	0.18	5.10	0.73	0/1410	0.00
	K86M	88th	3.42	86.20	0.00	90.50	-5.74	58th	0.00	0.00	0.00	0.00	0.18	5.10	0.73	0/1410	0.00
	F182A	89th	3.43	86.17	0.00	90.50	-5.74	59th	0.00	0.03	0.00	0.00	0.18	5.10	0.73	0/1427	0.00
	F182Y	89th	3.42	86.20	0.00	90.50	-5.74	59th	0.00	0.03	0.00	0.00	0.18	5.10	0.73	0/1427	0.00
	F182W	89th	3.42	86.20	0.00	90.50	-5.74	59th	0.00	0.03	0.00	0.00	0.18	5.10	0.73	0/1427	0.00

Color cutoffs: green (good), yellow (caution) and red (warning)

The green-to-yellow cutoffs are set from statistics or from properties of the validation methods.
The yellow-to-red cutoffs are not derived from properties of the validations, but are set from our intuition and experience to serve as guidlines for when structures become seriously troubled.

Supplementary Table 1. Validation of the homology models by MolProbity.
(Related to METHODS)
The homology of ABCG2 variants were generated by PyMOL and consequently evaluated using MolProbity to evaluate the reliable and quality of the 3D models. The cutoffs are categorized in to 3 levels, warning (red), caution (yellow) and good (green), respectively.

Ligand	Model	Method	Variants	ECL1								Valve				re-entry		ECL3									Docking score	Number of interacting residues
				D419	S420	T421	I423	Q424	N425	A427	G428	G553	L554	L555	N557	F578	Q582	F591	Y605	A606	T607	C608	E612	Y613	K616	Q617		
Mitoxantrone	6vxj	LigPlot+	WT		■/●		●	●/●		●			●						■	■/■	■/■			●/●	●/●		-7.8	16
		PyMOL	WT		●/●	●/●	●/●	●/●		●			●/●						●/●	●/●	■/■			■/●	●/●	●/●	-7.8	23
Pheophorbide A	6vxj	LigPlot+	WT		■/●	●	●	●/●				●	●		■/●				●/●	●/●	●/●			●/●	■/●	■/●	5.8	22
		PyMOL	WT		■/●	●/●	●/●	●/●		●			●/●		●/●	●	●		●/●	●/●	●/●		●	●/●	■/●	■/●	5.8	28
Daunorubicin	6vxj	LigPlot+	WT		■/●	●	●	●/●					●/●					●		●	■/●			●/●	●/●	●/●	-7.0	18
			R482G		■/●	●	●	●/●					●/●					●	●	●	■/●			●/●	●/●	●	-7.0	18
			F431Y		■/●	●	●	●/●					●/●					●	●	●	■/●			●/●	●/●	●	-6.9	18
		PyMOL	WT	●	●/●	●	●/●	●/●	●	●			●/●		●			●	●	●/●	■/●	●		●/●	●/●	●/●	-7.0	26
			R482G		●/●	●	●/●	●/●	●	●			●/●					●	●	●/●	■/●	●		●/●	●/●	●/●	-7.0	24
			F431Y		●/●	●	●/●	■/●	●	●			●/●		●			●	●	●/●	■/●	●		●/●	●/●	●/●	-6.9	25
Rhodamine 123	6vxj	LigPlot+	WT		■/●		●/●	●/●					●								●			●/●	■		-10.0	11
			R482G		■/■		●/●	●/●					●								●			●/●	■		-10.0	11
			F431Y		■/■		●/●	●/●					●								●			●/●	■		-10.0	11
			F431W		■/●		●/●	●/●					●								●			●/●	■		-10.0	11
			K86M		■/●		●/●	●/●					●								●			●/●	■		-10.0	11
		PyMOL	WT		■/●	●/●	●/●	●/●		●			●								●/●			●/●	●/●	●/●	-10.0	18
			R482G		■/●	●/●	●/●	●/●		●			●								●/●			●/●	●/●	●/●	-10.0	18
			F431Y		■/●	●/●	●/●	●/●		●			●								●/●			●/●	●/●	●/●	-10.0	18
			F431W		■/●	●/●	●/●	●/●		●			●								●/●			●/●	●/●	●/●	-10.0	18
			K86M		■/●	●/●	●/●	●/●		●			●						●		●/●			●/●	●/●	●/●	-10.0	19
Ko143	6vxj	LigPlot+	WT		●		●/●	●/●			●		●/●						●/●	●/●	■/■			●/●	●	●	-9.0	18
		PyMOL	WT		●/●		●/●	●/●		●			●/●	●				●	●/●	●/●	■/■			●/●	●	●	-9.0	21

● Hydrophobic Interaction (blue circle)
■ Polar Interaction (H-bond) (red square)

Supplementary Table 2. Interacting residues in the upper cavity.
(Related to Figure 7)
Docking of ABCG2 variants with their substrates and inhibitor at the upper cavity was analyzed using CB-Dock to identify the interacting residues with binding affinity score. The interacting residues were visualized by PyMOL and LigPlot+ and summarized in the table. Hydrophobic (non-polar) interactions are shown in blue circles while the hydrogen-bond (polar) interactions are indicated in red square. One symbol represents one interaction.

Mutants	Forward primer (5'->3')	Reverse primer (5'->3')
F182A	GGTTGGAACTCAGgcTATCCGTGGTGTG	CACACCACGGATAgcCTGAGTTCCAACC
F182Y	GGTTGGAACTCAGTaTATCCGTGGTGTG	CACACCACGGATAtACTGAGTTCCAACC
F182W	GGTTGGAACTCAGTggATCCGTGGTGTG	CACACCACGGATccACTGAGTTCCAACC
F431A	GCTGGGGTTCTCgcCTTCCTGACGACC	GGTCGTCAGGAAGgcGAGAACCCCAGC
F431Y	GCTGGGGTTCTCTaCTTCCTGACGACC	GGTCGTCAGGAAGtAGAGAACCCCAGC
F431W	GCTGGGGTTCTCTggTTCCTGACGACC	GGTCGTCAGGAAccAGAGAACCCCAGC
F432A	GGGGTTCTCTTCgcCCTGACGACCAACC	GGTTGGTCGTCAGGgcGAAGAGAACCCC
F432Y	GGGGTTCTCTTCTaCCTGACGACCAACC	GGTTGGTCGTCAGGtAGAAGAGAACCCC
F432W	GGGGTTCTCTTCTggCTGACGACCAACC	GGTTGGTCGTCAGccAGAAGAGAACCCC
F431A - F432A	GCTGGGGTTCTCgcCgcCCTGACGACCAACC	GGTTGGTCGTCAGGgcGgcGAGAACCCCAGC
F431Y - F432Y	GCTGGGGTTCTCTaCTaCCTGACGACCAACC	GGTTGGTCGTCAGGtAGtAGAGAACCCCAGC
F431W - F432W	GCTGGGGTTCTCTggTggCTGACGACCAACC	GGTTGGTCGTCAGccAccAGAGAACCCCAGC
T435A	CTTCTTCCTGACGgCCAACCAGTGTTTC	GAAACACTGGTTGGcCGTCAGGAAGAAG
T435G	CTTCTTCCTGACGggCAACCAGTGTTTC	GAAACACTGGTTGccCGTCAGGAAGAAG
T435L	CTTCTTCCTGACGctCAACCAGTGTTTC	GAAACACTGGTTGagCGTCAGGAAGAAG
T435S	CTTCTTCCTGACGtCCAACCAGTGTTTC	GAAACACTGGTTGGaCGTCAGGAAGAAG
T435D	CTTCTTCCTGACGgaCAACCAGTGTTTC	GAAACACTGGTTGtcCGTCAGGAAGAAG
N436A	CTTCCTGACGACCgCCCAGTGTTTCAGC	GCTGAAACACTGGGcGGTCGTCAGGAAG
N436L	CTTCCTGACGACCctCCAGTGTTTCAGC	GCTGAAACACTGGagGGTCGTCAGGAAG
N436F	CTTCCTGACGACCttCCAGTGTTTCAGC	GCTGAAACACTGGaaGGTCGTCAGGAAG
N436Q	CTTCCTGACGACCcAgCAGTGTTTCAGC	GCTGAAACACTGcTgGGTCGTCAGGAAG
N436K	CTTCCTGACGACCAAgCAGTGTTTCAGC	GCTGAAACACTGcTTGGTCGTCAGGAAG
T435G - N436A	CTCTTCTTCCTGACGggCgCCCAGTGTTTCAGC	GCTGAAACACTGGGcGccCGTCAGGAAGAAGAG
T435G - N436L	CTCTTCTTCCTGACGgCCctCCAGTGTTTCAGC	GCTGAAACACTGGagGGcCGTCAGGAAGAAGAG
T435L - N436F	CTCTTCTTCCTGACGctCttCCAGTGTTTCAGC	GCTGAAACACTGGaaGagCGTCAGGAAGAAGAG

Supplementary Table 3. Oligonucleotide primers used to generate ABCG2 mutations
(Related to METHODS)
